# Supplementary material for: Targeted Isolation of Prenylated Flavonoids from Paulownia tomentosa Fruit Extracts via AI-Guided Workflow Integrating LC-UV-HRMS/MS
Source: Metabolites. 2025 Sep 17;15(9):616. doi: 10.3390/metabo15090616 (PMC12471768; doi:10.3390/metabo15090616)
Supplement: Supplementary file 1 [file metabolites-15-00616-s001.zip › metabolites-3847810-supplementary/Supplementary Material File S2.pdf]

Supplementary material 2

Targeted Isolation of Prenylated Flavonoids from *Paulownia tomentosa* Fruit Extracts via AI-Guided Workflow Integrating LC-UV-HRMS/MS

Tomas Rypar [1,2], Lenka Molcanova [3], Barbora Valkova [3], Ema Hromadkova [3], Christoph Bueschl [2], Bernhard Seidl [2,4], Karel Smejkal [3] and Rainer Schuhmacher [2,*]

[1] Department of Chemistry and Biochemistry, Mendel University in Brno, Zemedelska 1, CZ 613 00 Brno, Czech Republic; tomas.rypar@mendelu.cz (T.R.)
[2] Institute of Bioanalytics and Agro-Metabolomics, Department of Agricultural Sciences, BOKU University, Konrad-Lorenz-Str. 20, 3430 Tulln, Austria; christoph.bueschl@boku.ac.at (C.B.); bernhard.seidl@boku.ac.at (B.S.)
[3] Department of Natural Drugs, Masaryk University, Palackeho 1946/1, CZ 612 00 Brno, Czech Republic; molcanoval@pharm.muni.cz (L.M.); 527417@mail.muni.cz (B.V.); 524487@mail.muni.cz (E.H.); smejkalk@pharm.muni.cz (K.S.)
[4] Core Facility Bioactive Molecules: Screening and Analysis, BOKU University, Konrad-Lorenz-Str. 20, 3430 Tulln, Austria
[*] Correspondence: rainer.schuhmacher@boku.ac.at

Table of Content:

Figure S1 Separation scheme 3
Figure S2 Extracted ion chromatograms of coeluting features with ID 4115 and 4104 4
Figure S3 Fragmentation spectra of the targeted feature ID: 8830........ 6
Figure S4 Fragmentation spectra of the targeted feature ID: 15466........ 7
Figure S5 Fragmentation spectra of the targeted feature ID: 15912........ 8
Figure S6 Fragmentation spectra of the targeted feature ID: 13504........ 9
Figure S7 Fragmentation spectra of the targeted feature ID: 14313........ 10
Figure S8 HPLC chromatogram at 280 nm with UV spectrum of 6-prenyl-4′-O-methyltaxifolin (**1**)..... 11
Figure S9 EIC and HRMS spectra of 6-prenyl-4′-*O*-methyltaxifolin (**1**)........ 12
Figure S10 1H NMR spectrum (400 MHz, CDCl3) of 6-prenyl-4′-O-methyltaxifolin (**1**) 13
Figure S11 Detail on 1H NMR and HSQC spectra (400 MHz) of 6-prenyl-4′-O-methyltaxifolin (**1**) measured in DMSO-d_6 vs. CDCl3 14
Figure S12 HSQC spectrum (400 MHz, CDCl3) of 6-prenyl-4′-O-methyltaxifolin (**1**)........ 15
Figure S13 HMBC spectrum (400 MHz, CDCl3) of 6-prenyl-4′-O-methyltaxifolin (**1**)........ 16
Figure S14 COSY spectrum (400 MHz, CDCl3) of 6-prenyl-4′-O-methyltaxifolin (**1**) 17
Figure S15 NOESY spectrum (400 MHz, CDCl3) of 6-prenyl-4′-O-methyltaxifolin (**1**)........ 18
Figure S16 HPLC chromatogram at 280 nm with UV spectrum of 3′-O-methyldiplacone (**2**)........ 19
Figure S17 EIC and HRMS spectra of 3′-*O*-methyldiplacone (**2**)........ 20
Figure S18 1H NMR spectrum (400 MHz, DMSO-d_6) of 3′-O-methyldiplacone (**2**)........ 21
Figure S19 HSQC spectrum (400 MHz, DMSO-d_6) of 3′-O-methyldiplacone (**2**) 22
Figure S20 HMBC spectrum (400 MHz, DMSO-d_6) of 3′-O-methyldiplacone (**2**) 23
Figure S21 COSY spectrum (400 MHz, DMSO-d_6) of 3′-O-methyldiplacone (**2**)........ 24
Figure S22 NOESY spectrum (400 MHz, DMSO-d_6) of 3′-O-methyldiplacone (**2**)........ 25
Figure S23 HPLC chromatogram at 280 nm with UV spectrum of 3′,4′-O-dimethyldiplacone (**3**)........ 26

Figure S24 EIC and HRMS spectra of 3′,4′-O-dimethyldiplacone (**3**) 27
Figure S25 1H NMR spectrum (400 MHz, DMSO-d_6) of 3′,4′-O-dimethyldiplacone (**3**) 28
Figure S26 HSQC spectrum (400 MHz, DMSO-d_6) of 3′,4′-O-dimethyldiplacone (**3**) 29
Figure S27 HMBC spectrum (400 MHz, DMSO-d_6) of 3′,4′-O-dimethyldiplacone (**3**) 30
Figure S28 COSY spectrum (400 MHz, DMSO-d_6) of 3′,4′-O-dimethyldiplacone (**3**) 31
Figure S29 NOESY spectrum (400 MHz, DMSO-d_6) of 3′,4′-O-dimethyldiplacone (**3**) 32
Figure S30 HPLC chromatogram at 280 nm with UV spectrum of tomentodiplacone M (**4**) 33
Figure S31 EIC and HRMS spectra of tomentodiplacone M (**4**) 34
Figure S32 1H NMR spectrum (400 MHz, DMSO-d_6) of tomentodiplacone M (**4**) 35
Figure S33 HSQC spectrum (400 MHz, DMSO-d_6) of tomentodiplacone M (**4**) 36
Figure S34 HMBC spectrum (400 MHz, DMSO-d_6) of tomentodiplacone M (**4**) 37
Figure S35 COSY spectrum (400 MHz, DMSO-d_6) of tomentodiplacone M (**4**) 38
Figure S36 NOESY spectrum (400 MHz, DMSO-d_6) of tomentodiplacone M (**4**) 39
Figure S37 HPLC chromatogram at 280 nm with UV spectrum of 3′,4′-O-dimethylpaulodiplacone A (**5**) 40
Figure S38 EIC and HRMS spectra of 3′,4′-*O*-dimethylpaulodiplacone A (**5**) 41
Figure S39 1H NMR spectrum (400 MHz, DMSO-d_6) of 3′,4′-O-dimethylpaulodiplacone A (**5**) 42
Figure S40 HSQC spectrum (400 MHz, DMSO-d_6) of 3′,4′-O-dimethylpaulodiplacone A (**5**) 43
Figure S41 HMBC spectrum (400 MHz, DMSO-d_6) of 3′,4′-O-dimethylpaulodiplacone A (**5**) 44
Figure S42 COSY spectrum (400 MHz, DMSO-d_6) of 3′,4′-O-dimethylpaulodiplacone A (**5**) 45
Figure S43 NOESY spectrum (400 MHz, DMSO-d_6) of 3′,4′-O-dimethylpaulodiplacone A (**5**) 46

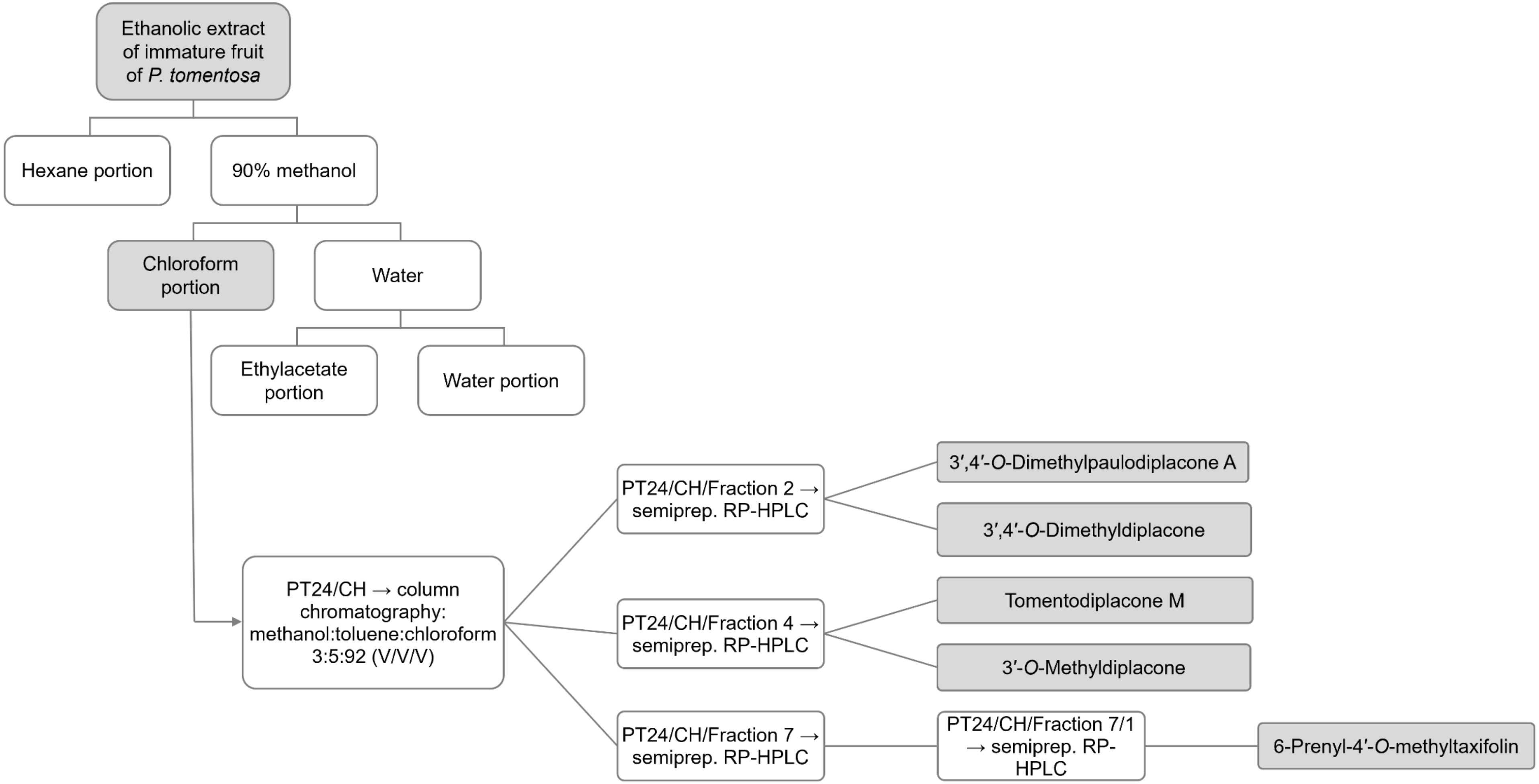

Figure S1. Separation scheme starting with liquid-liquid extraction and chloroform chromatographic separation on silica gel for the isolation of prenylated/geranylated flavonoids from unripe fruits of *Paulownia tomentosa*

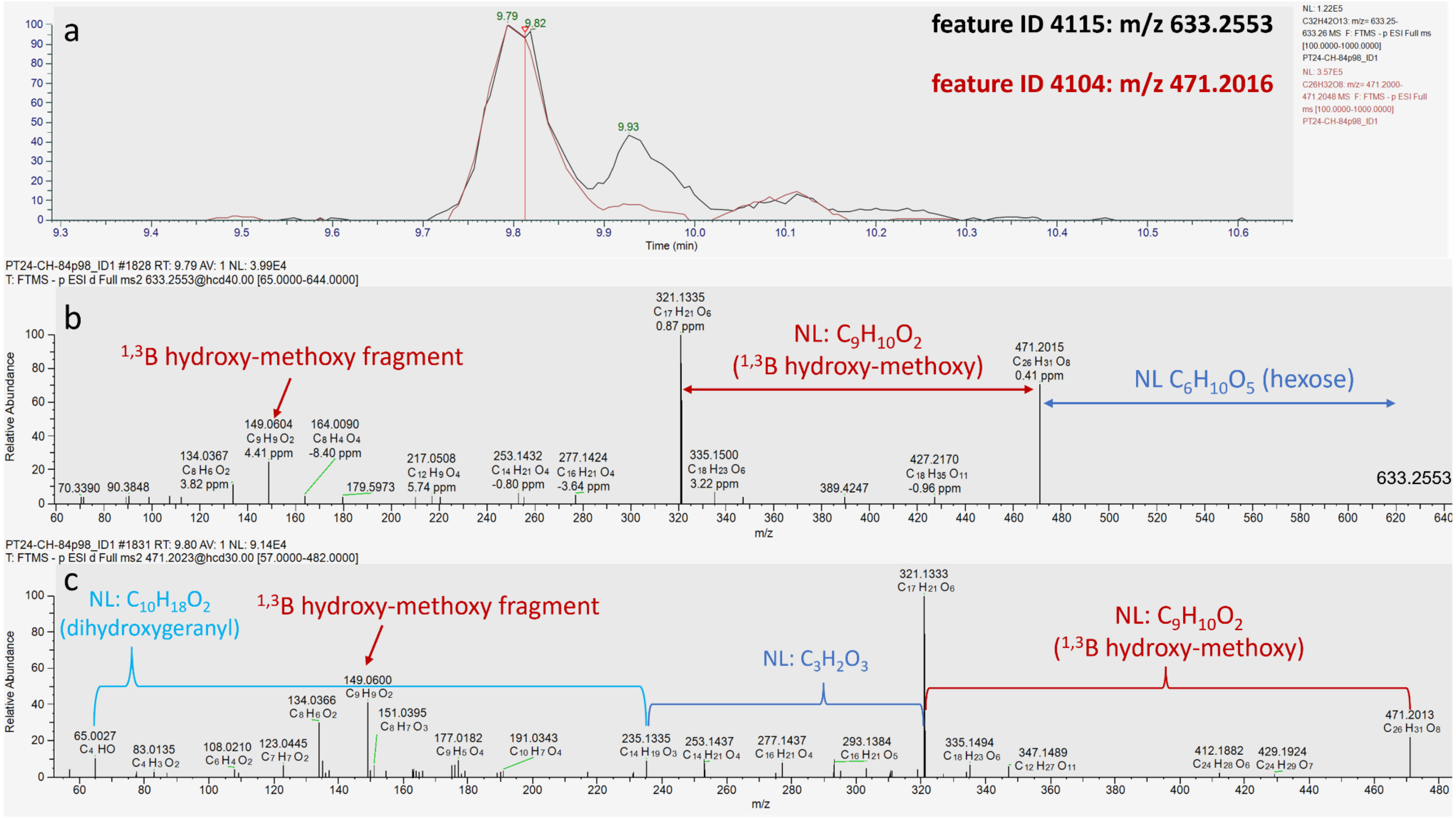

Figure S2. Extracted ion chromatograms of coeluting features with ID 4115 and 4104 indicating in-source fragmentation (a). Fragmentation spectra of the feature ID 4115 using HCD 40 eV, detected in negative ionization mode at m/z 633.2553 and RT 9.8 in fraction 1 3 (b), indicating presence of glycosylated flavonoid with hydroxy-methoxy groups on ring B. Fragmentation spectra of perfectly coeluting feature ID 4104 at *m/z* 471.2015 using HCD 30 eV, confirming presence of geranylated flavonoid with hydroxy-methoxy groups on ring B (c)

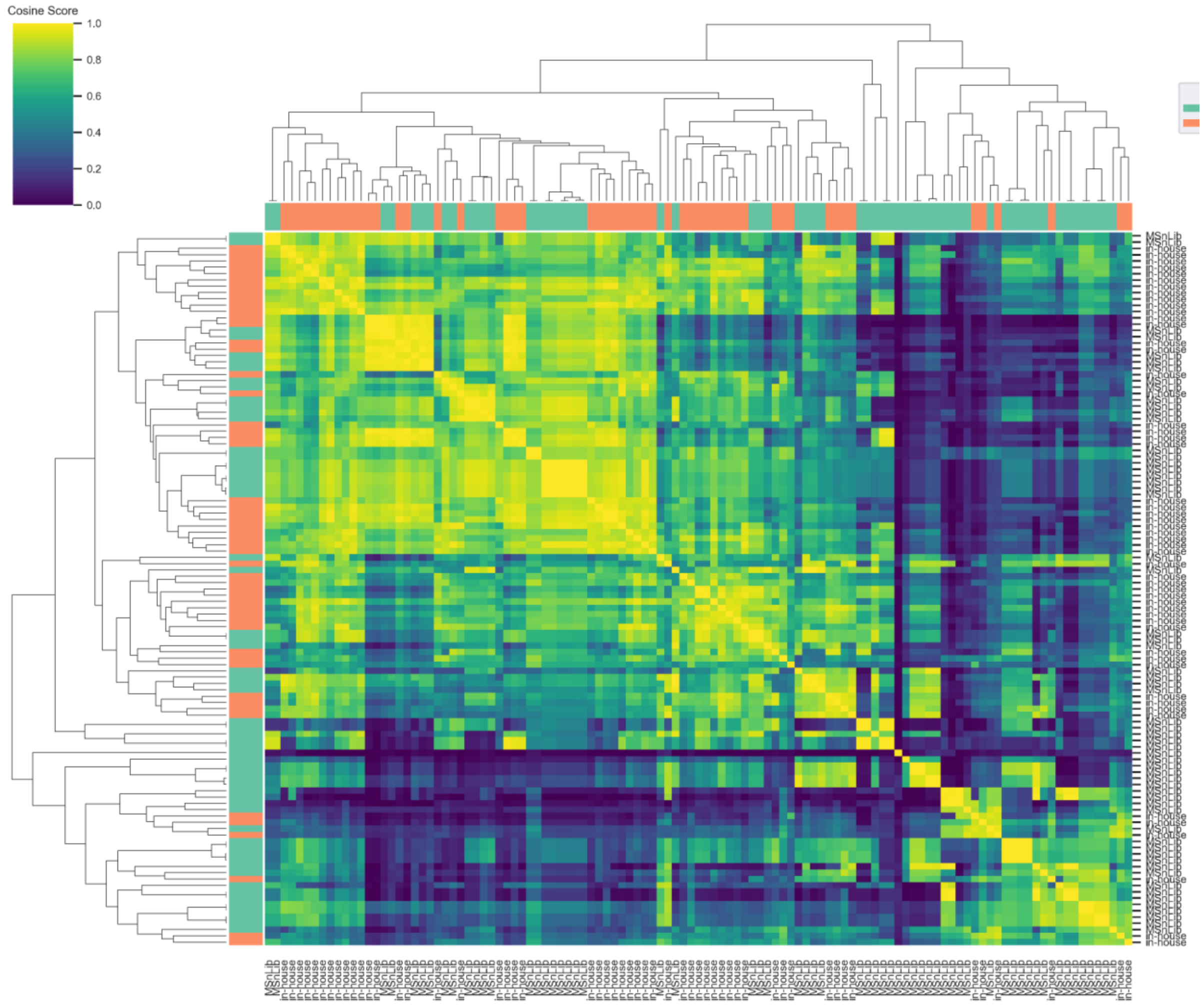

Figure S3. Comparison of MS/MS spectra similarity of our in-house library with public MSnLib using negative ionization mode, indicating a high similarity and overlap of some compounds

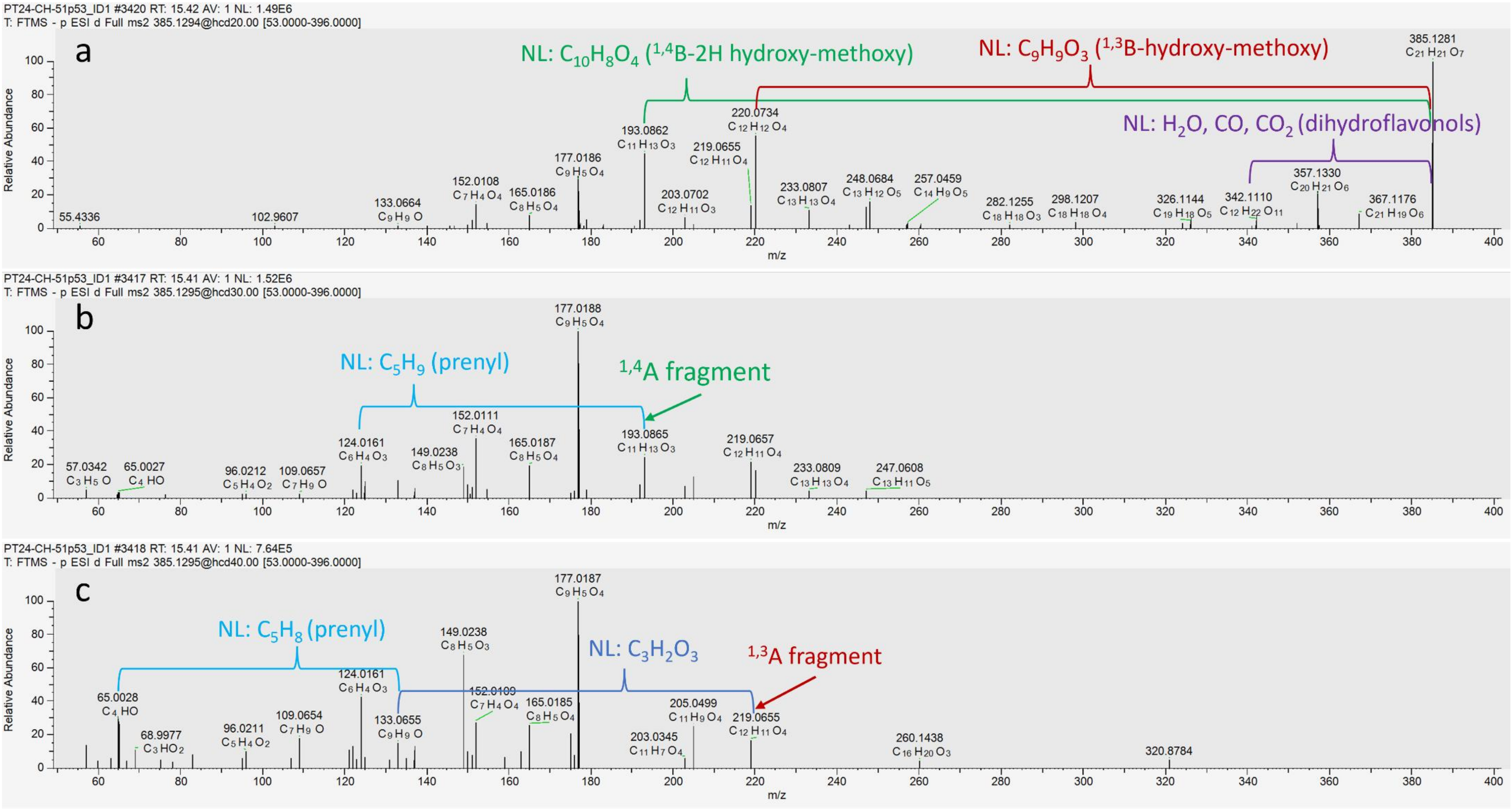

Figure S3. Fragmentation spectra of the targeted feature ID: 8830, with *m/z* 385.1294 and RT 15.37 most abundant in fraction 7, indicating presence of prenylated dihydroflavonol with hydroxy-methoxy groups on ring B and dihydroxy on the ring A. MS/MS spectra were acquired with collision energy HCD 20 eV (a), HCD 30 eV (b) and HCD 40 eV (c)

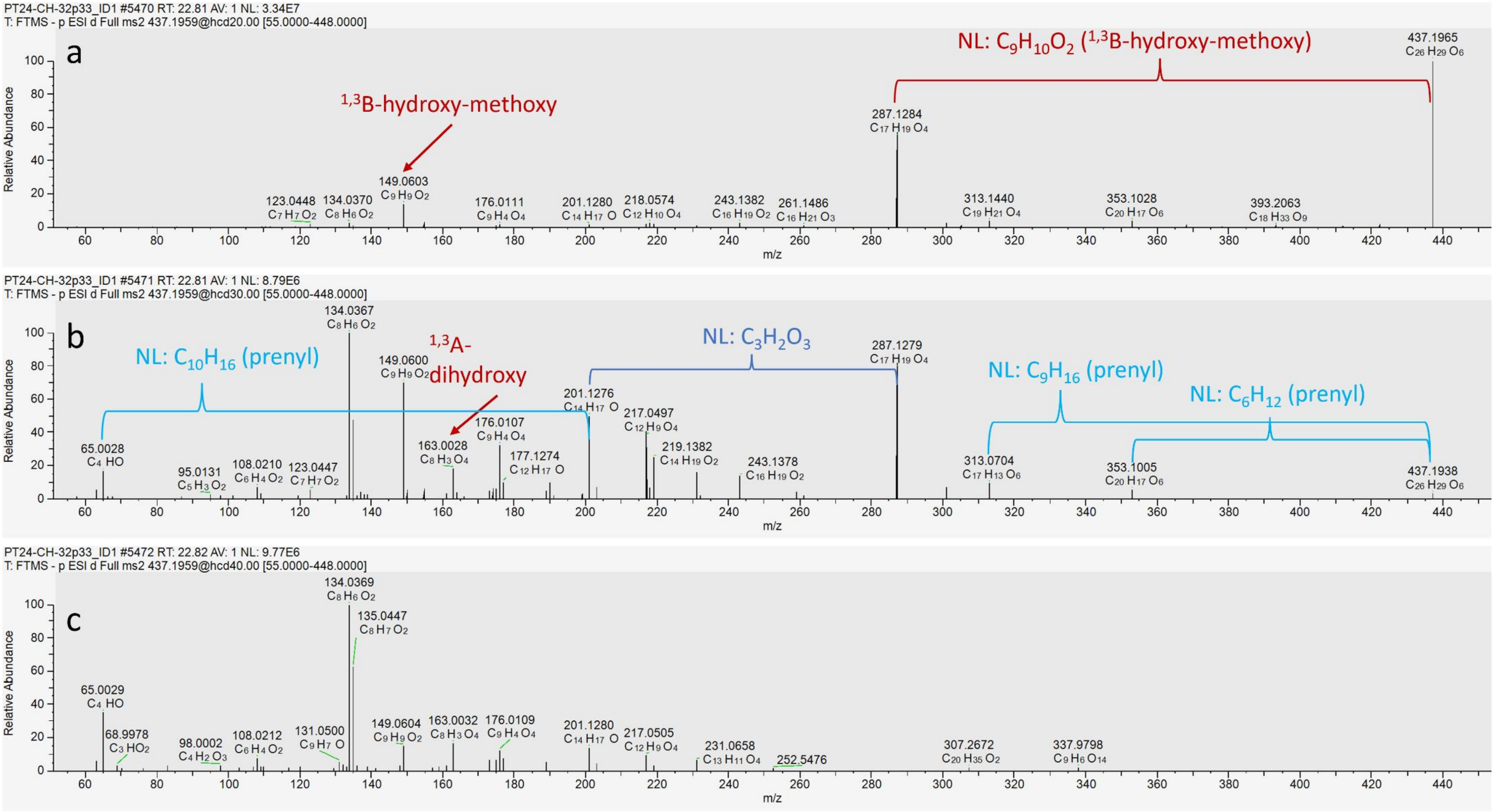

Figure S4. Fragmentation spectra of the targeted feature ID: 15466, with *m/z* 437.1967 and RT 22.77 most abundant in fraction 4, indicating presence of geranylated flavanone with hydroxy-methoxy groups on ring B and dihydroxy on the ring A. MS/MS spectra were acquired with collision energy HCD 20 eV (a), HCD 30 eV (b) and HCD 40 eV (c)

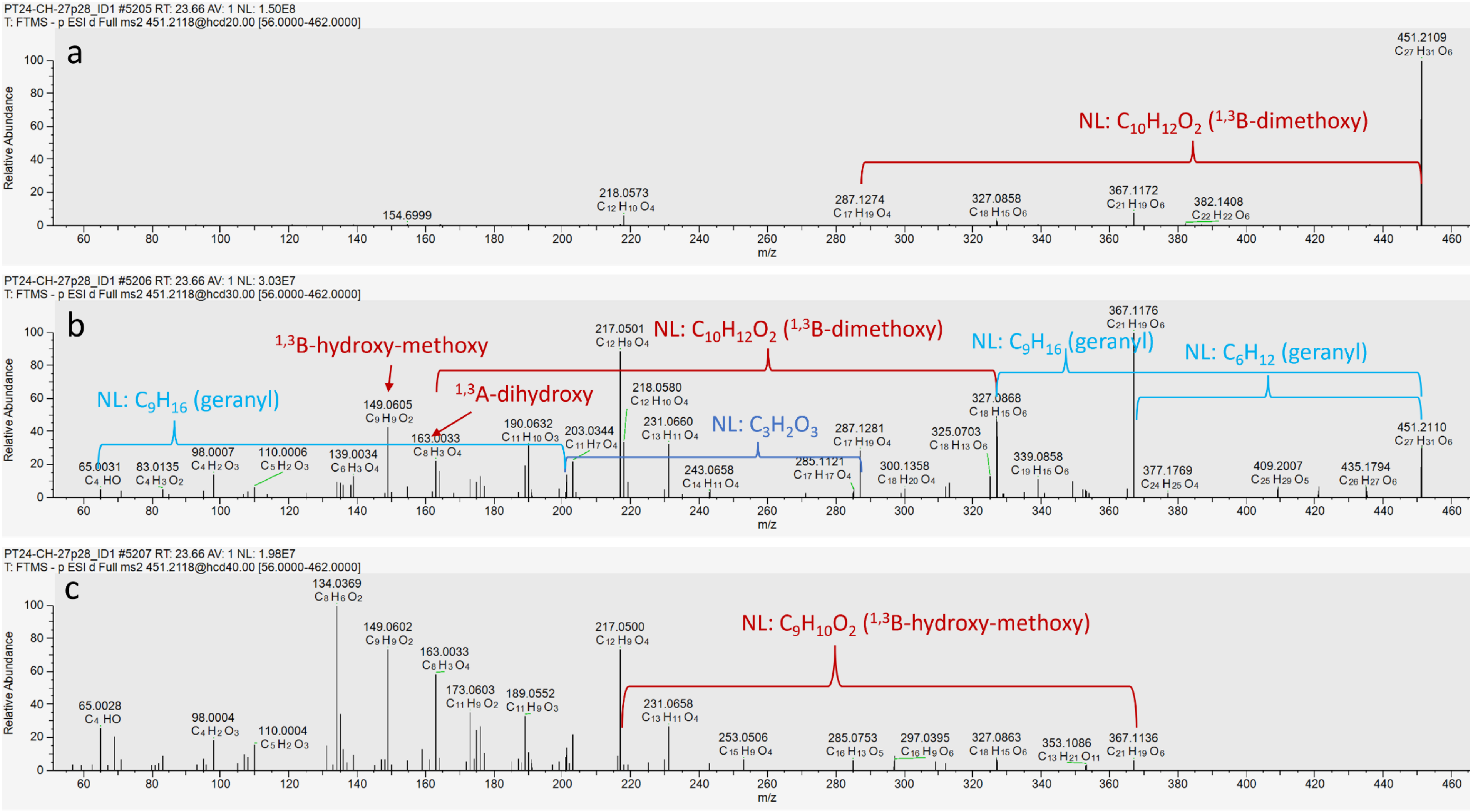

Figure S5. Fragmentation spectra of the targeted feature ID: 15912, with *m/z* 451.2125 and RT 23.66 most abundant in fraction 2, indicating presence of geranylated flavanone with dimethoxy groups on ring B and dihydroxy on the ring A. MS/MS spectra were acquired with collision energy HCD 20 eV (a), HCD 30 eV (b) and HCD 40 eV (c)

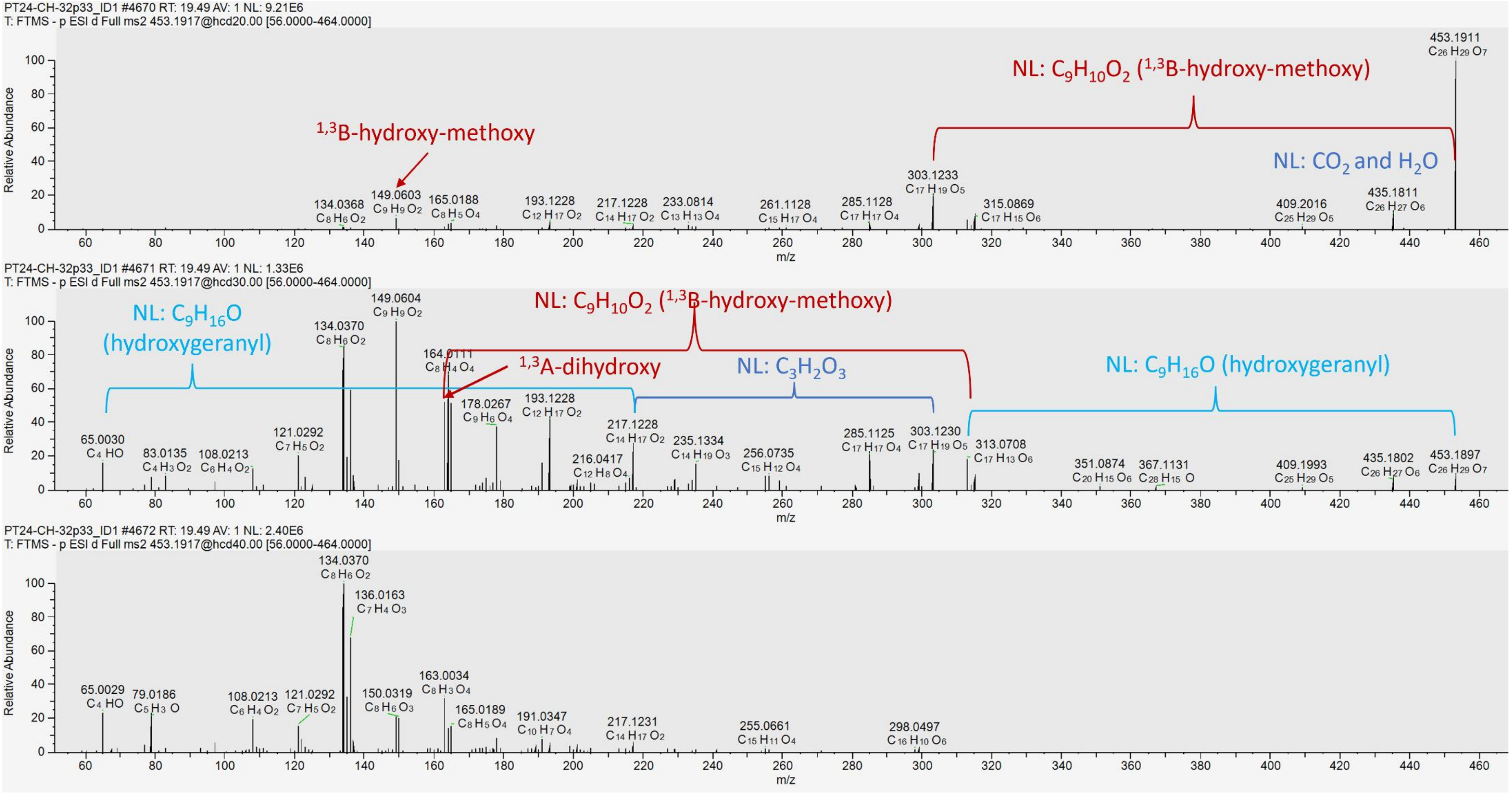

Figure S6. Fragmentation spectra of the targeted feature ID: 13504, with *m/z* 451.2125 and RT 23.66 most abundant in fraction 2, indicating presence of hydroxygeranylated flavanone with hydroxy-methoxy groups on ring B and dihydroxy on the ring A. MS/MS spectra were acquired with collision energy HCD 20 eV (a), HCD 30 eV (b) and HCD 40 eV (c)

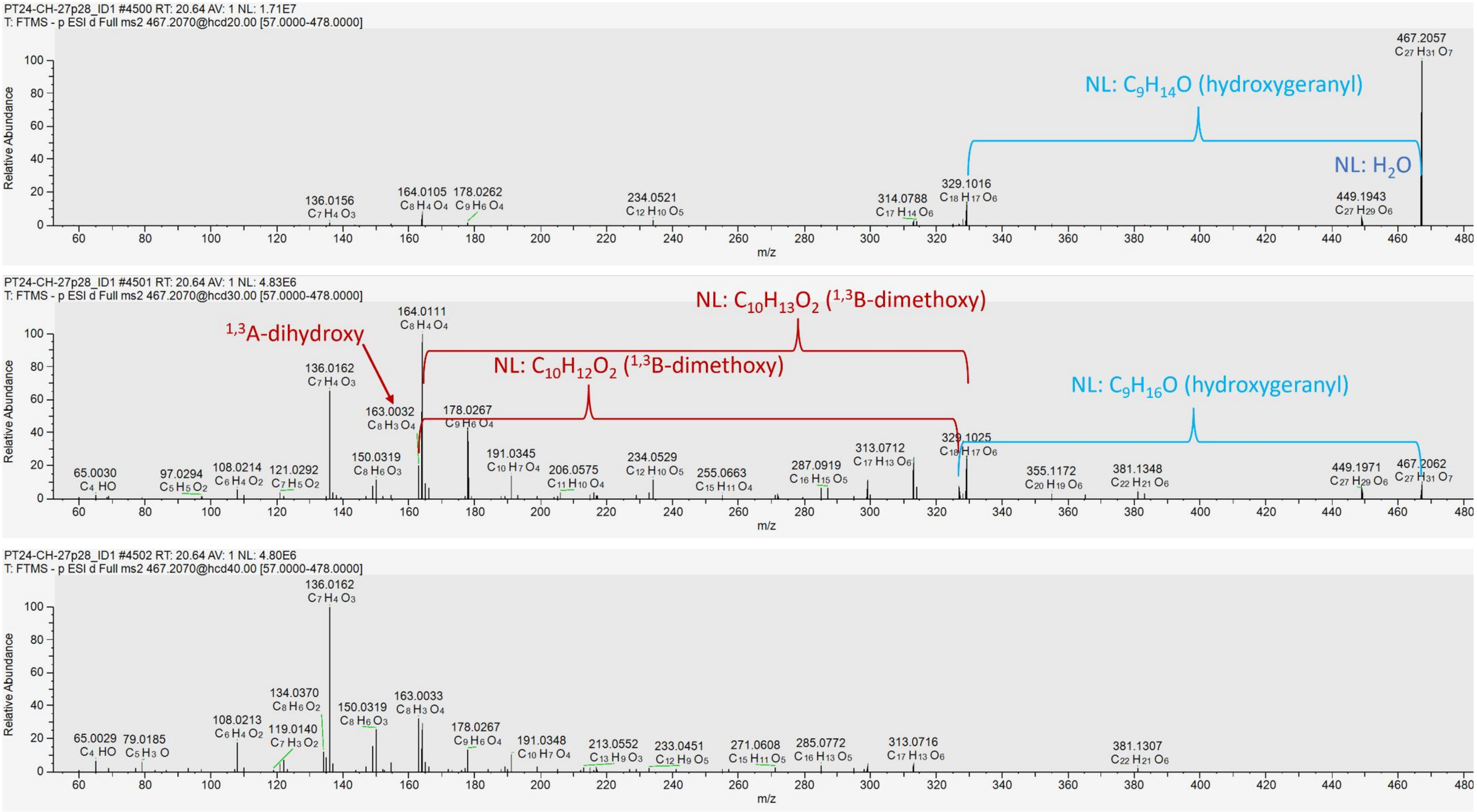

Figure S7. Fragmentation spectra of the targeted feature ID: 14313, with *m/z* 451.2125 and RT 23.66 most abundant in fraction 2, indicating presence of hydroxygeranylated flavanone with dimethoxy groups on ring B and dihydroxy on the ring A. MS/MS spectra were acquired with collision energy HCD 20 eV (a), HCD 30 eV (b) and HCD 40 eV (c)

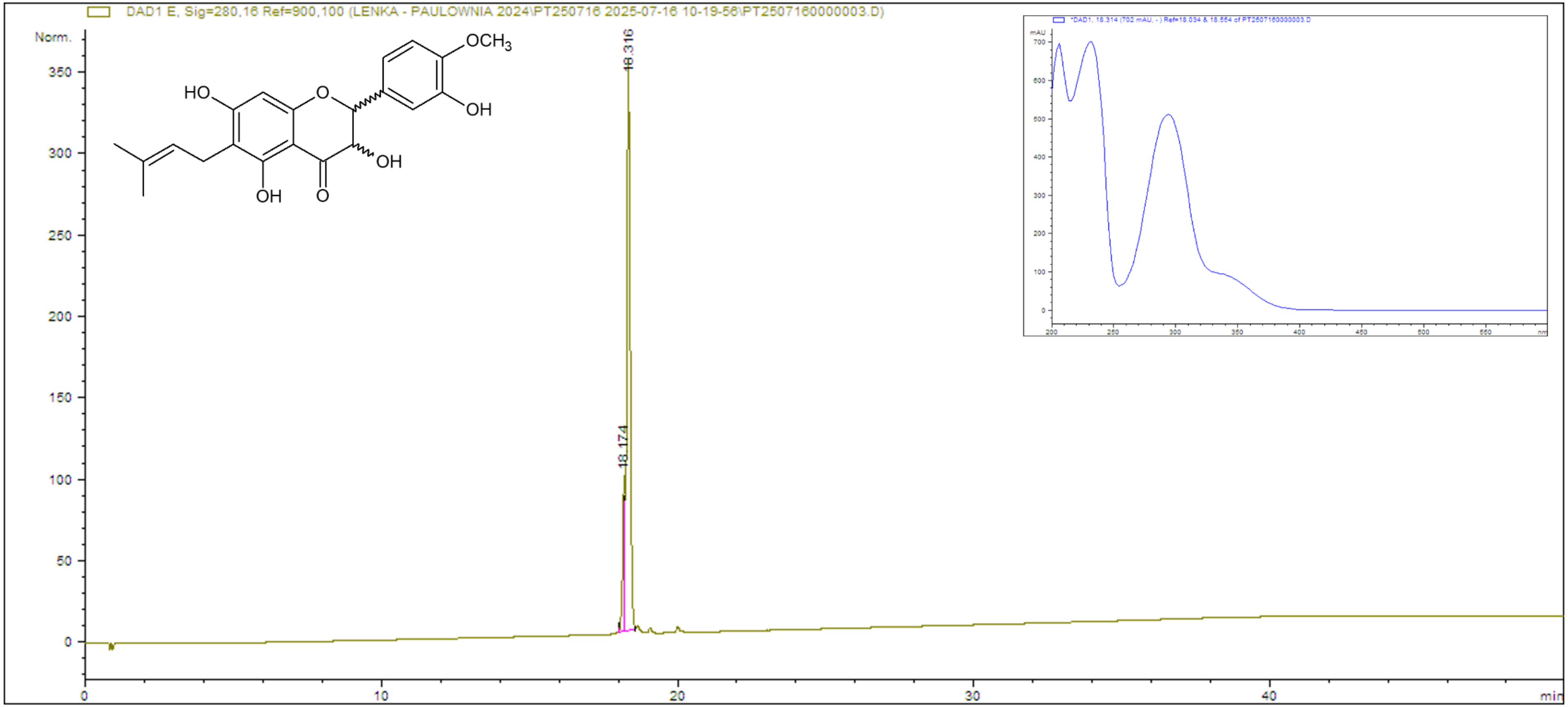

Figure S8. HPLC chromatogram at 280 nm with UV spectrum of 6-prenyl-4’-O-methyltaxifolin (**1**)

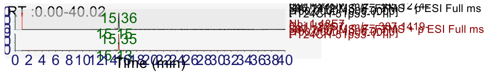

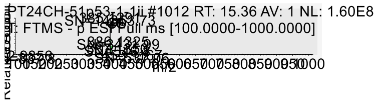

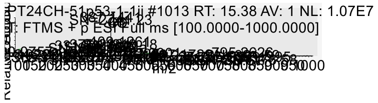

Figure S9. Extracted ion chromatogram (EIC) of the isolated compound **1** from fraction 7 with ID 8830 within 5 ppm *m/z* tolerance and 1% threshold for plot annotations in both negative (upper) and positive polarities (bottom). HRMS spectra at the peak apex in negative (upper) and positive ionization mode (bottom). Identified as 6-prenyl-4′-*O*-methyltaxifolin (**1**)

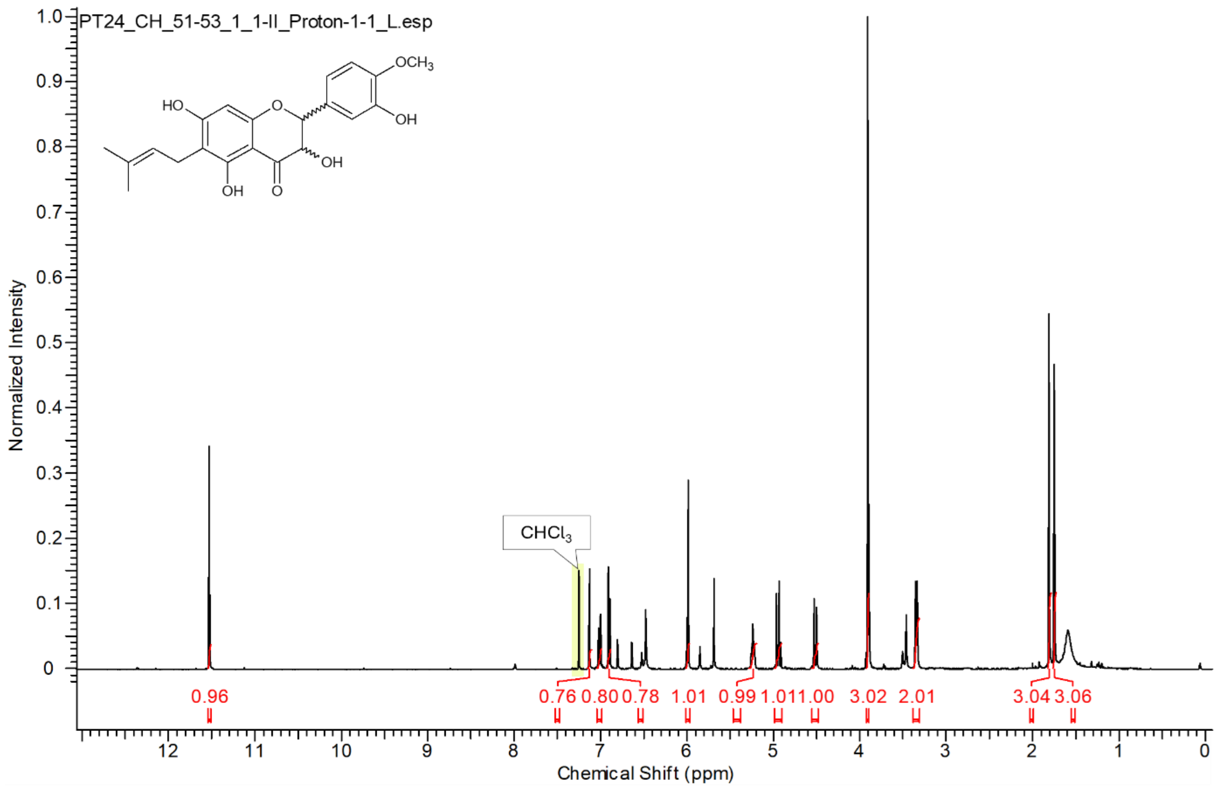

Figure S10. 1H NMR spectrum (400 MHz, CDCl3) of 6-prenyl-4’-O-methyltaxifolin (**1**)

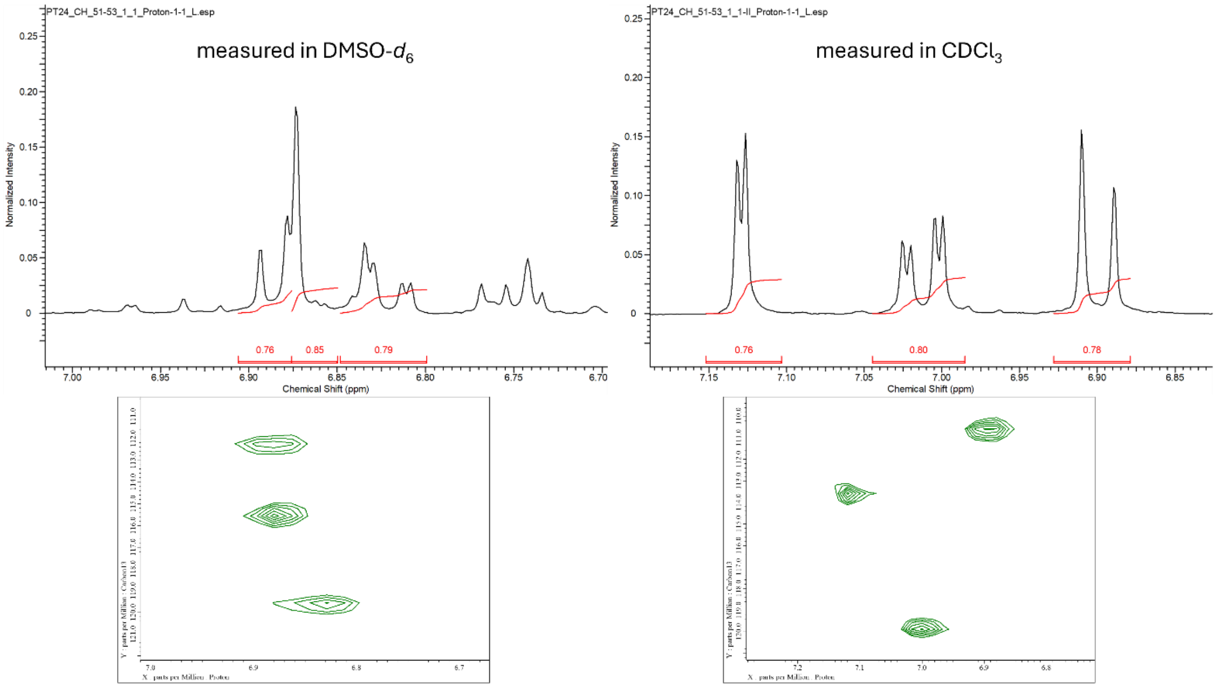

Figure S11. Detail on 1H NMR and HSQC spectra (400 MHz) of 6-prenyl-4′-O-methyltaxifolin (**1**) measured in DMSO-d_6 vs. CDCl3

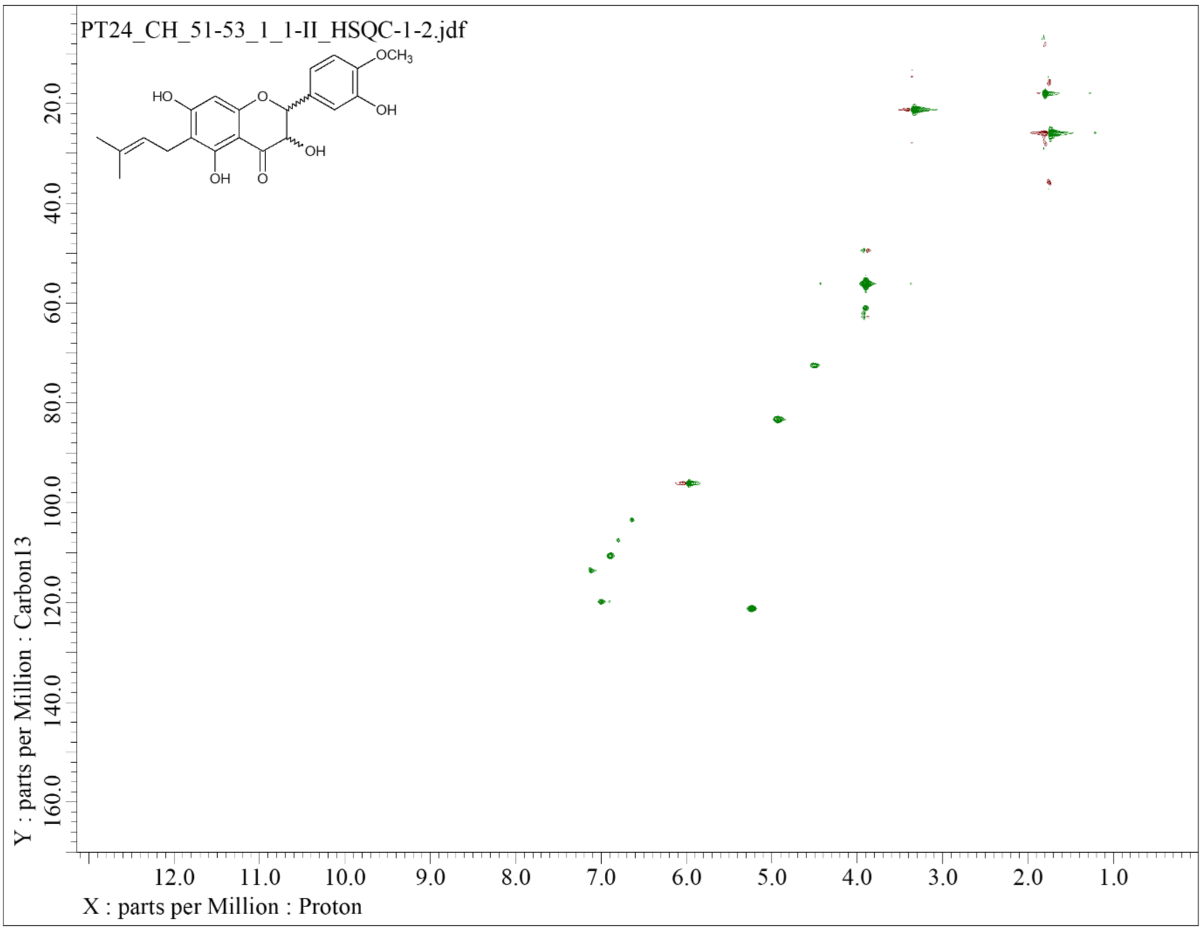

Figure S12. HSQC spectrum (400 MHz, CDCl3) of 6-prenyl-4′-O-methyltaxifolin (**1**)

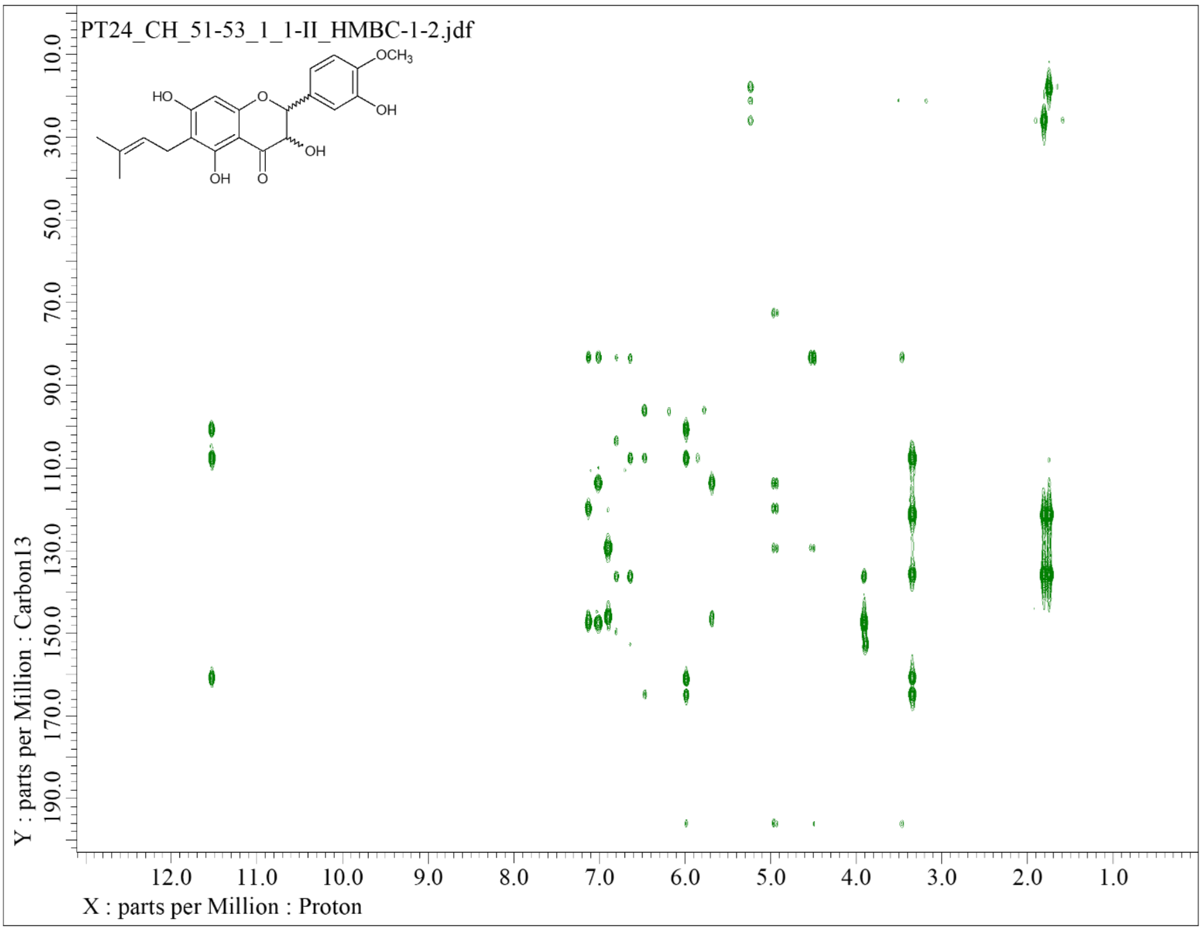

Figure S13. HMBC spectrum (400 MHz, CDCl3) of 6-prenyl-4'-O-methyltaxifolin (**1**)

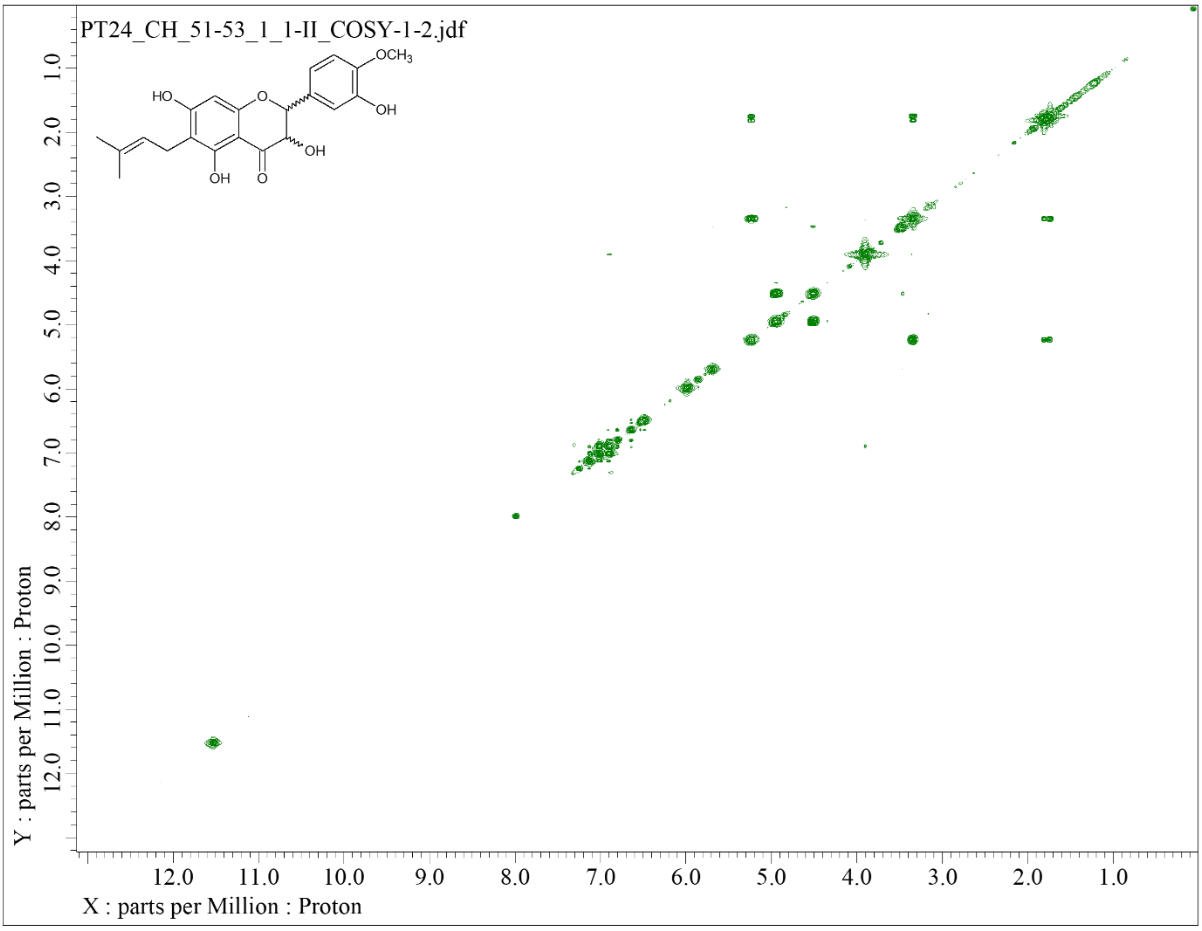

Figure S14. COSY spectrum (400 MHz, CDCl3) of 6-prenyl-4'-O-methyltaxifolin (**1**)

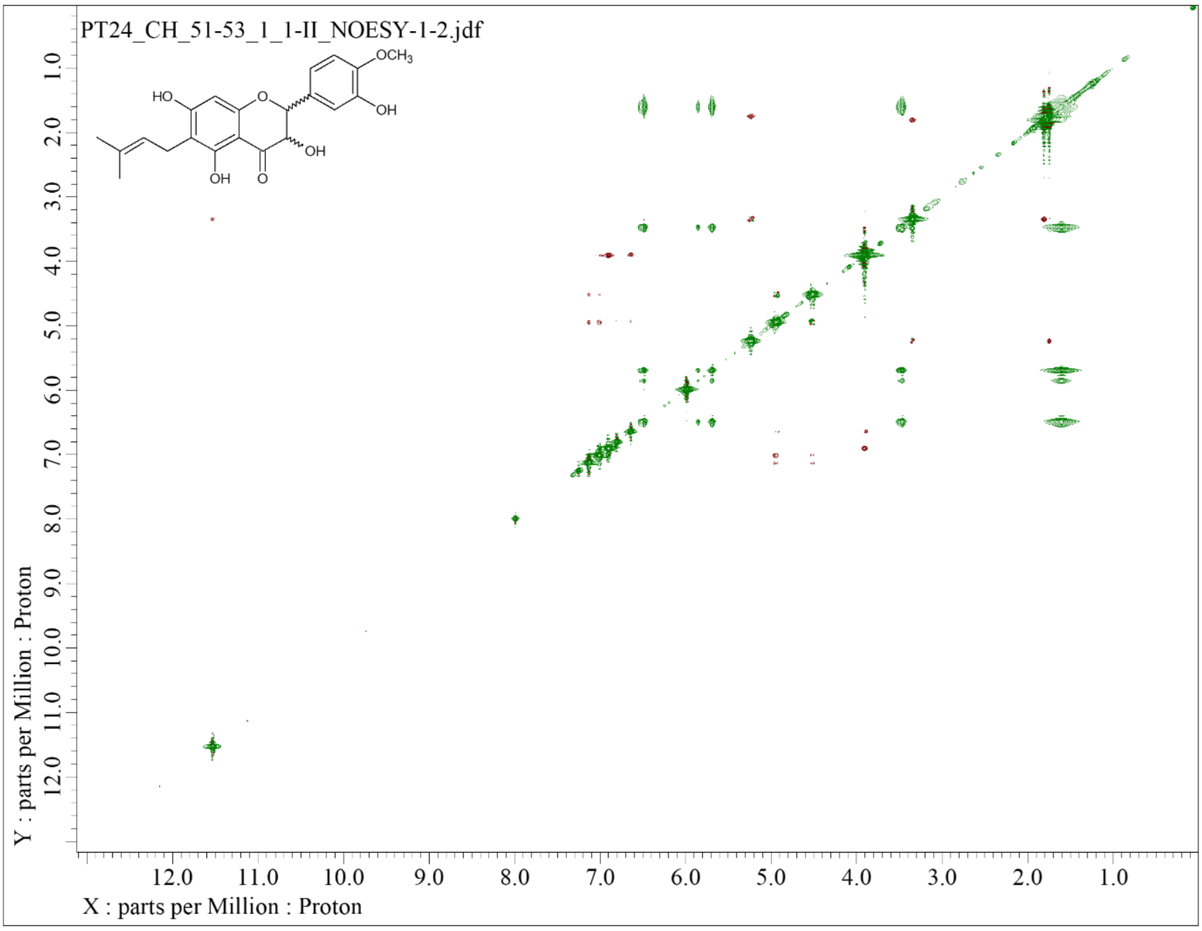

Figure S15. NOESY spectrum (400 MHz, CDCl3) of 6-prenyl-4′-O-methyltaxifolin (**1**)

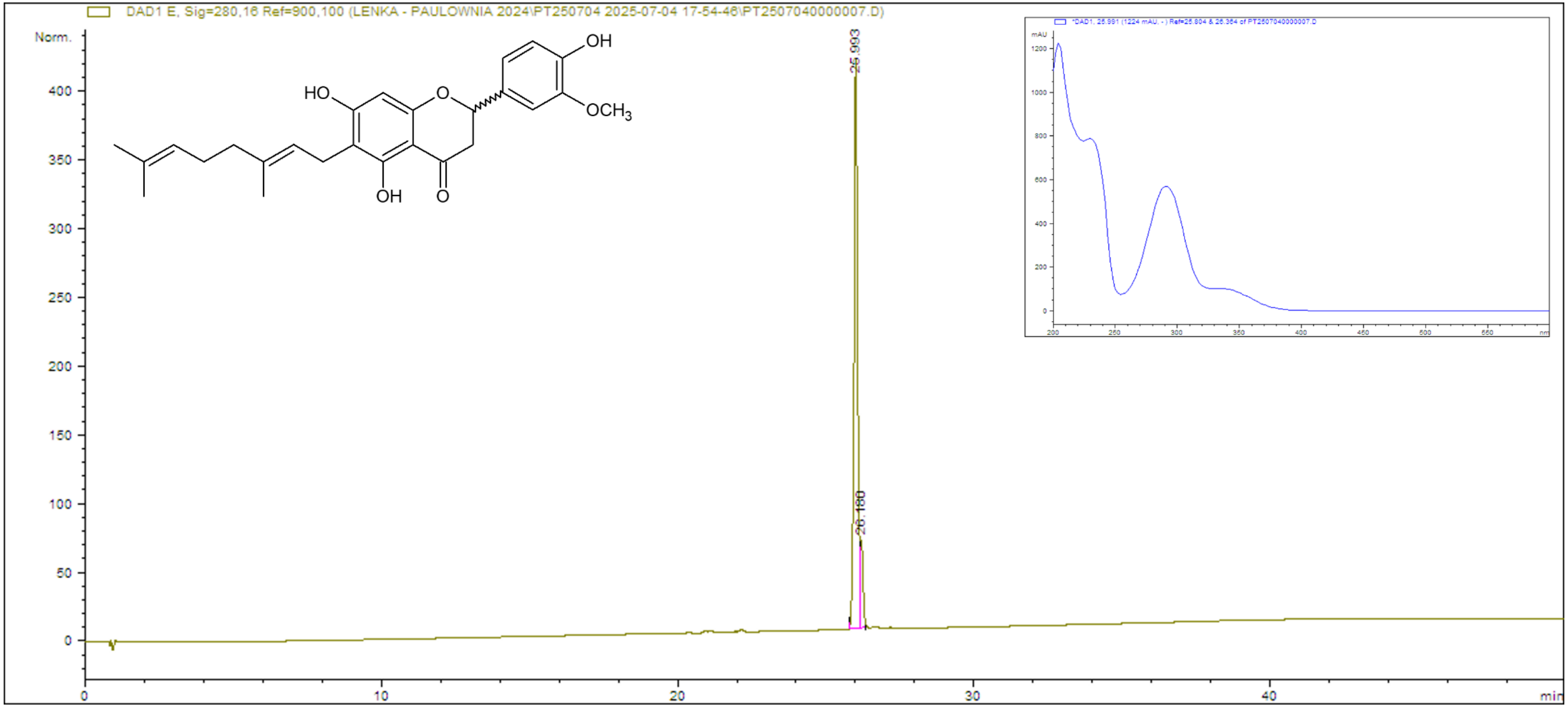

Figure S16. HPLC chromatogram at 280 nm with UV spectrum of 3'-O-methyldiplacone (**2**)

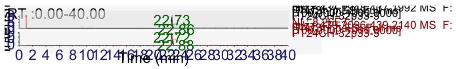

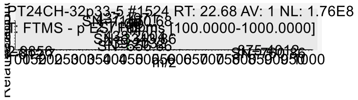

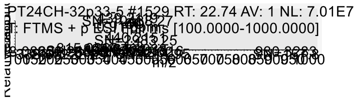

Figure S17. Extracted ion chromatogram (EIC) of the isolated compound **2** from fraction 4 with ID 15466 within 5 ppm *m/z* tolerance and 1% threshold for plot annotations in both negative (upper) and positive polarities (bottom). HRMS spectra at the peak apex in negative (upper) and positive ionization mode (bottom). Identified as 3′-*O*-methyldiplacone (**2**)

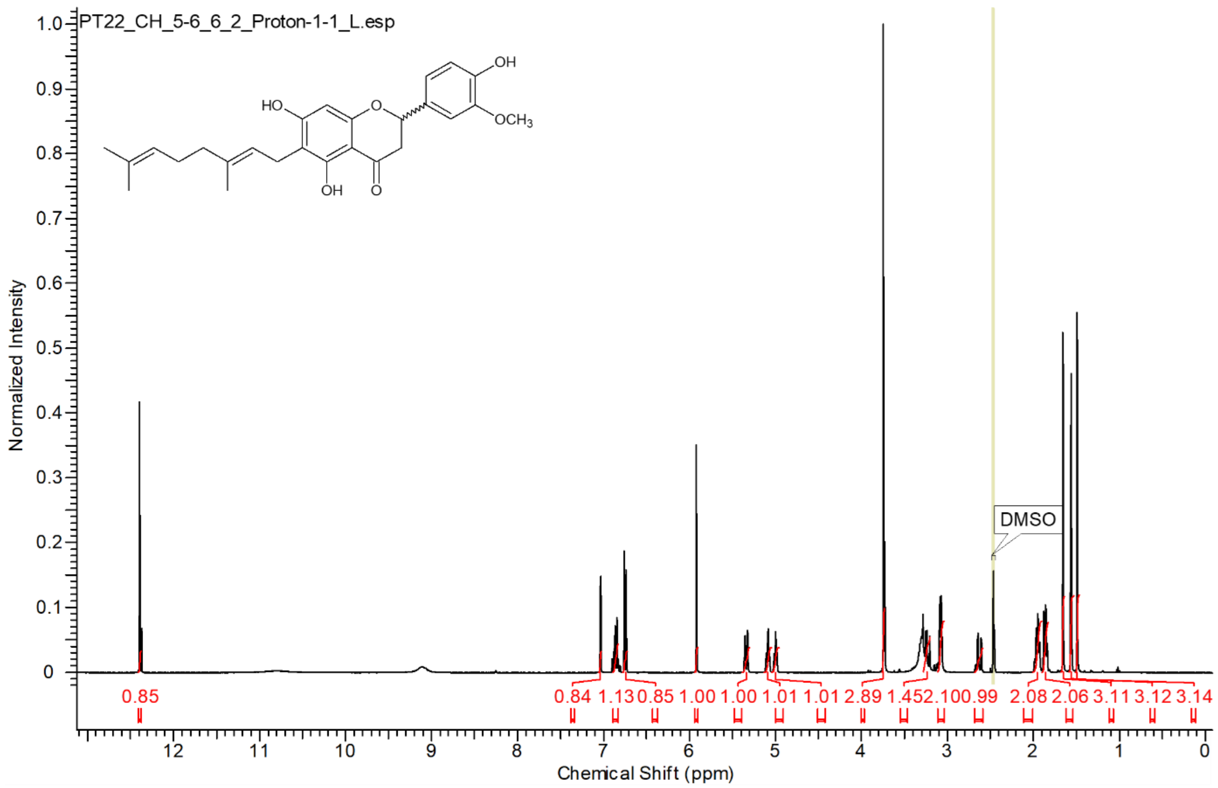

Figure S18. 1H NMR spectrum (400 MHz, DMSO-d_6) of 3'-O-methyldiplacone (**2**)

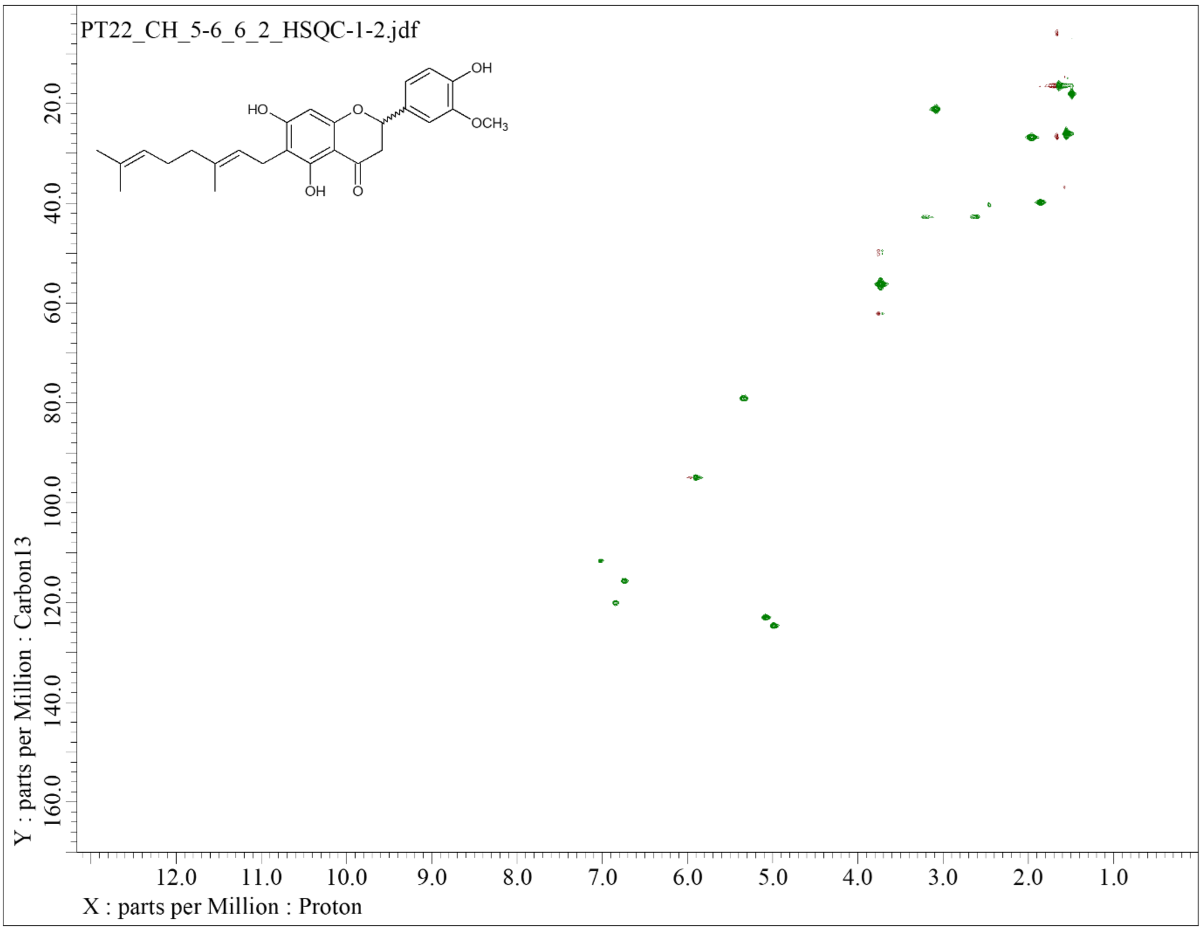

Figure S19. HSQC spectrum (400 MHz, DMSO-d$_6$) of 3′-O-methyldiplacone (**2**)

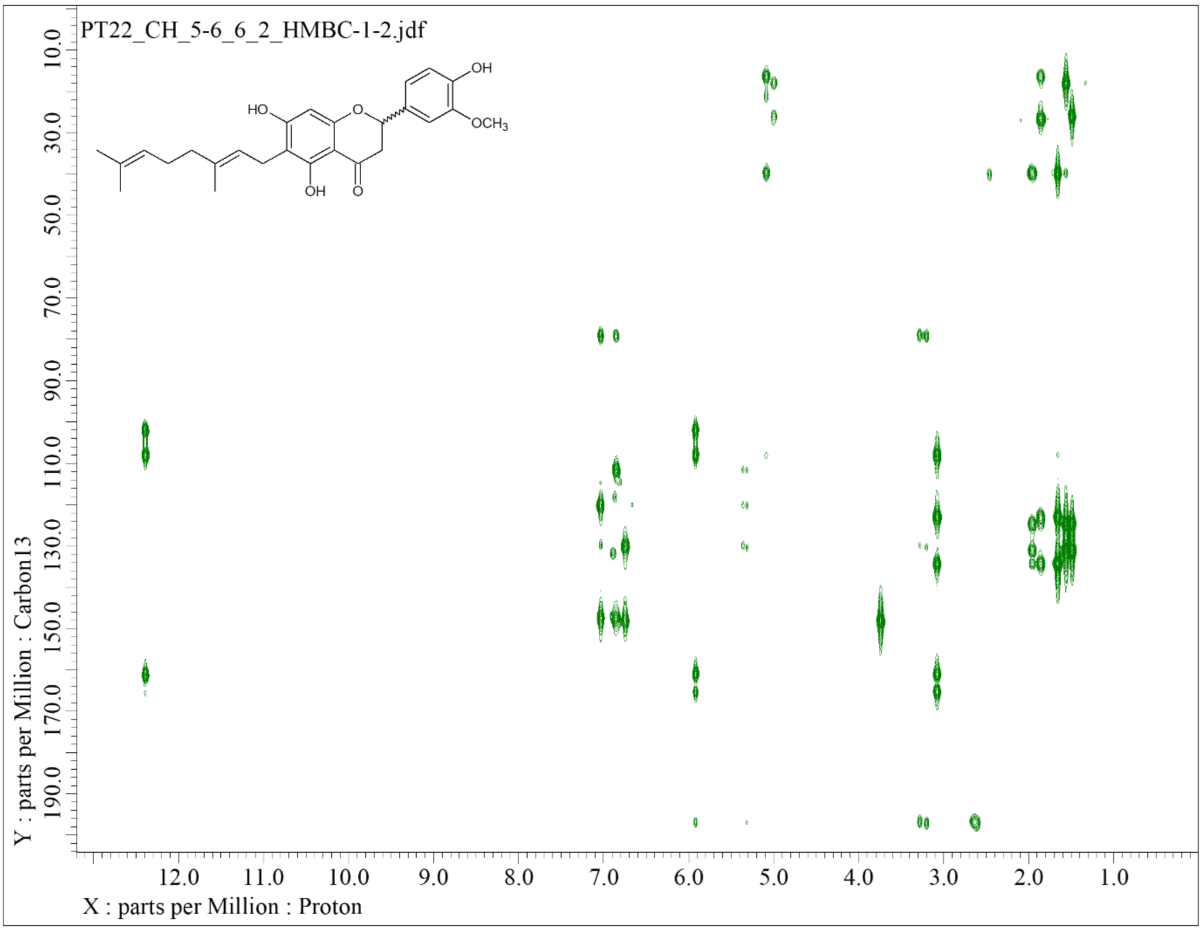

Figure S20. HMBC spectrum (400 MHz, DMSO-d_6) of 3′-O-methyldiplacone (**2**)

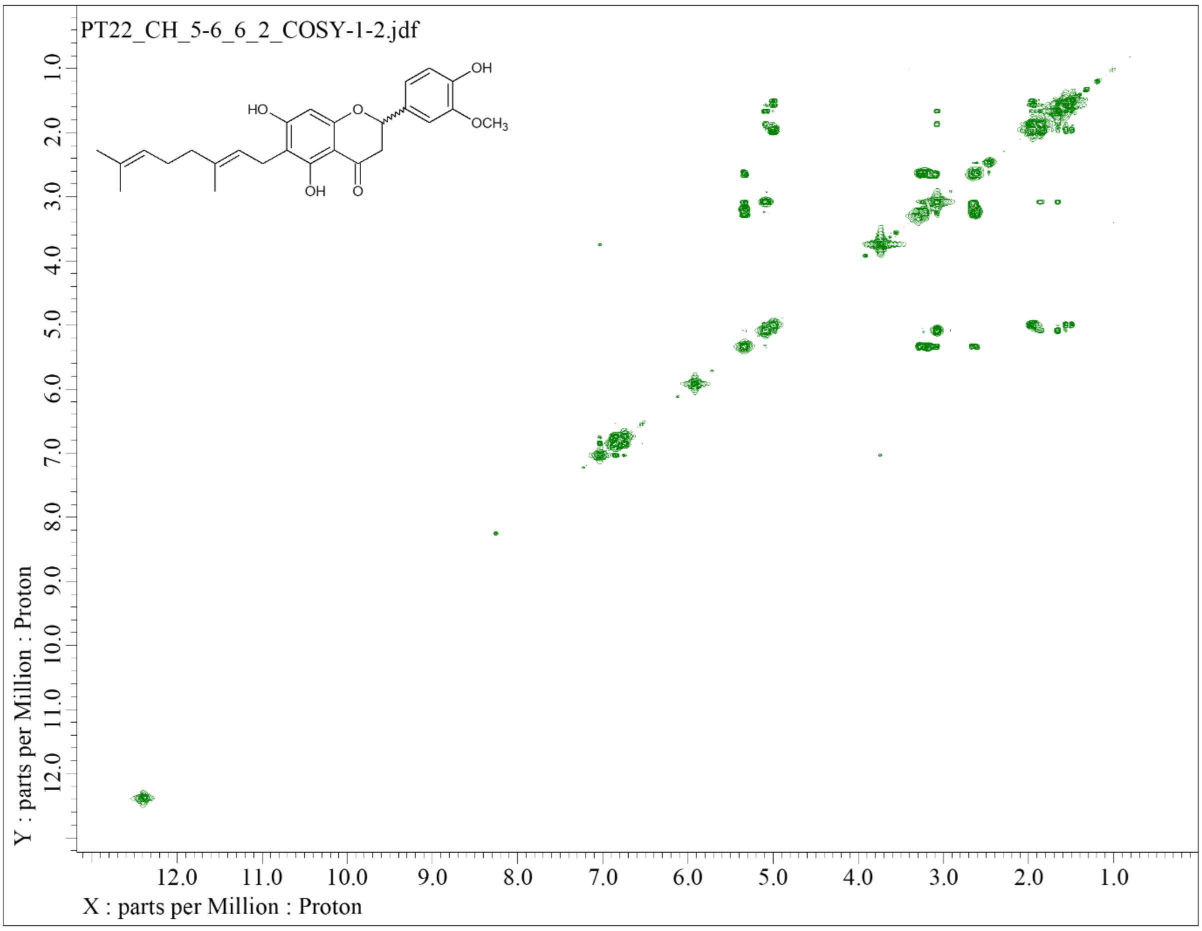

Figure S21. COSY spectrum (400 MHz, DMSO-d$_6$) of 3′-O-methyldiplacone (**2**)

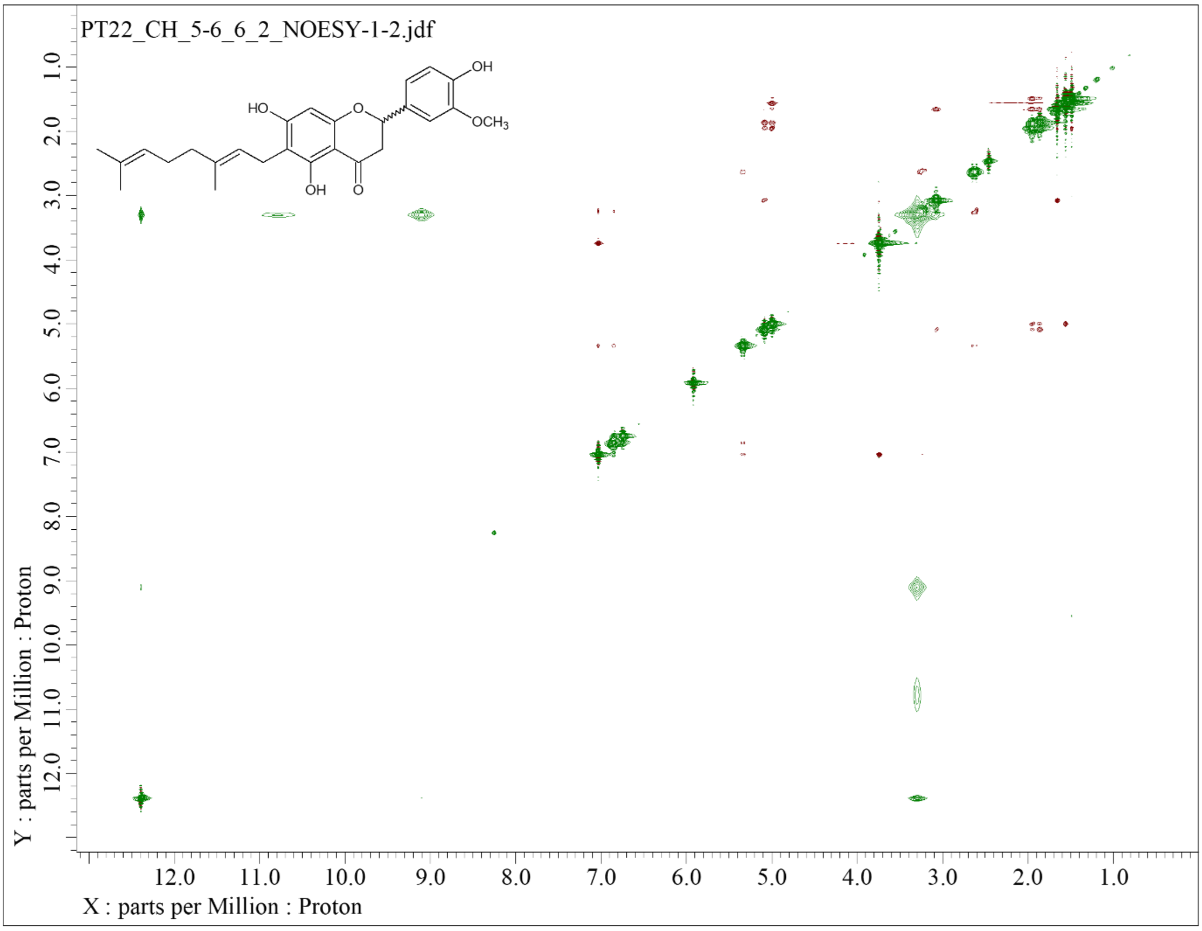

Figure S22. NOESY spectrum (400 MHz, DMSO-d_6) of 3′-O-methyldiplacone (**2**)

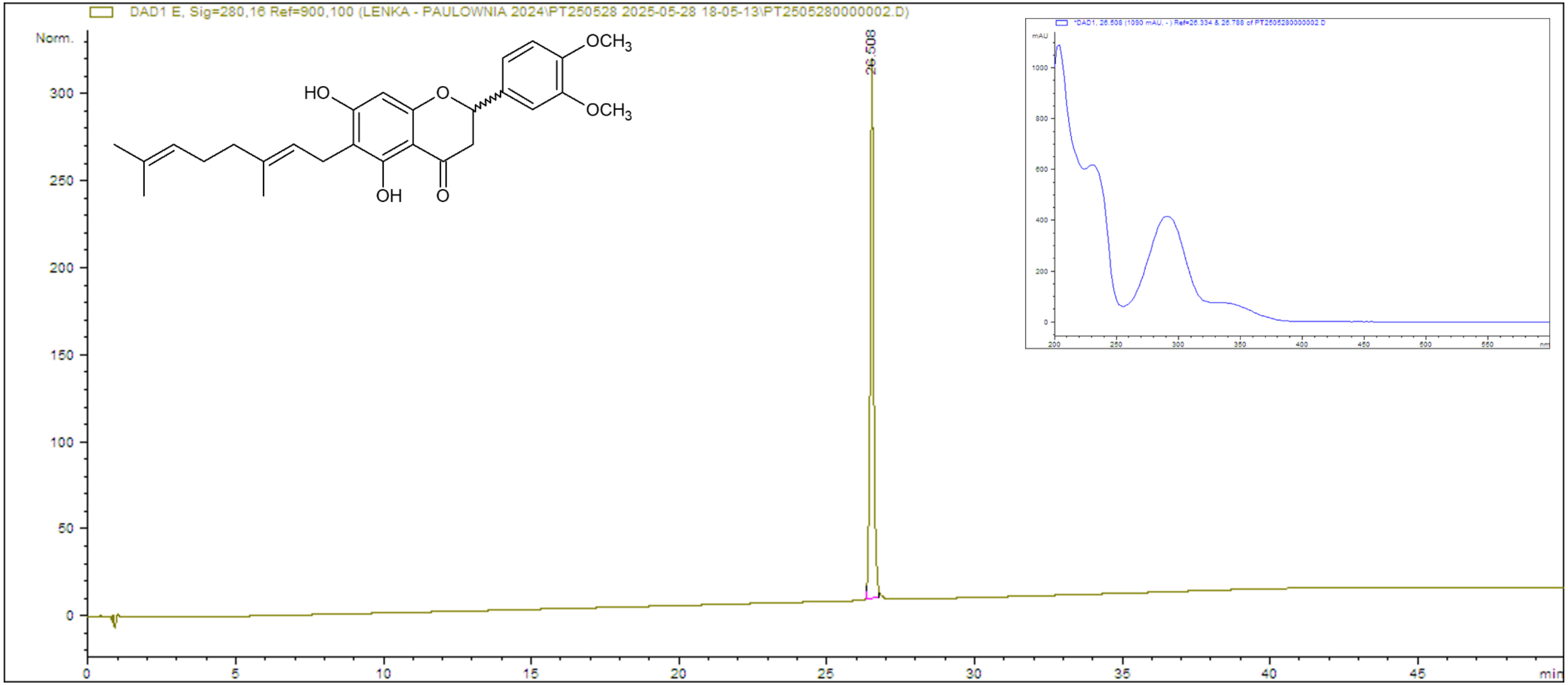

Figure S23. HPLC chromatogram at 280 nm with UV spectrum of 3’,4’-O-dimethyldiplacone (**3**)

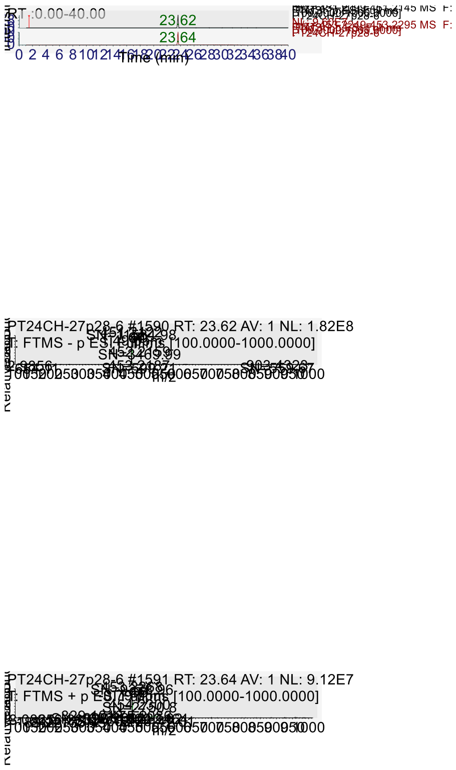

Figure S24. Extracted ion chromatogram (EIC) of the isolated compound **3** from fraction 2 with ID 15912 within 5 ppm m/z tolerance and 1% threshold for plot annotations in both negative (upper) and positive polarities (bottom). HRMS spectra at the peak apex in negative (upper) and positive ionization mode (bottom). Identified as 3′,4′-O-dimethyldiplacone (**3**)

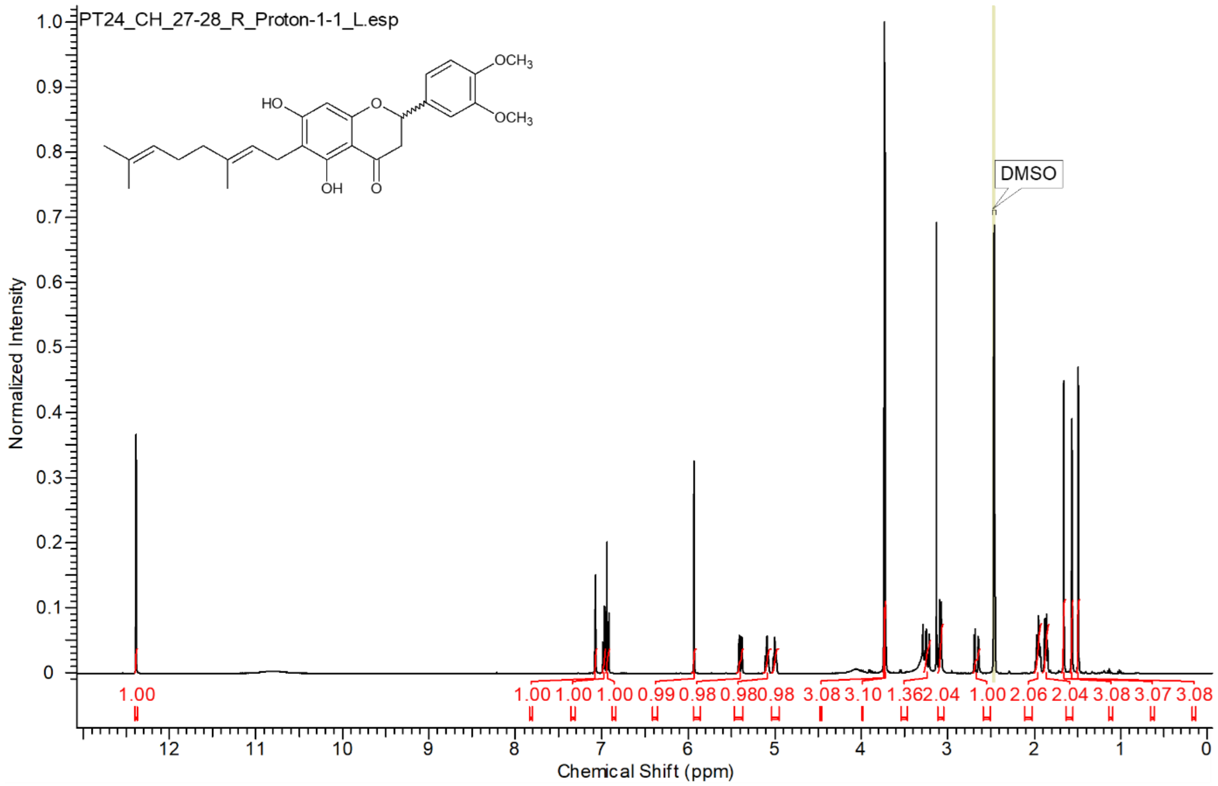

Figure S25. 1H NMR spectrum (400 MHz, DMSO-d_6) of 3′,4′-O-dimethyldiplacone (**3**)

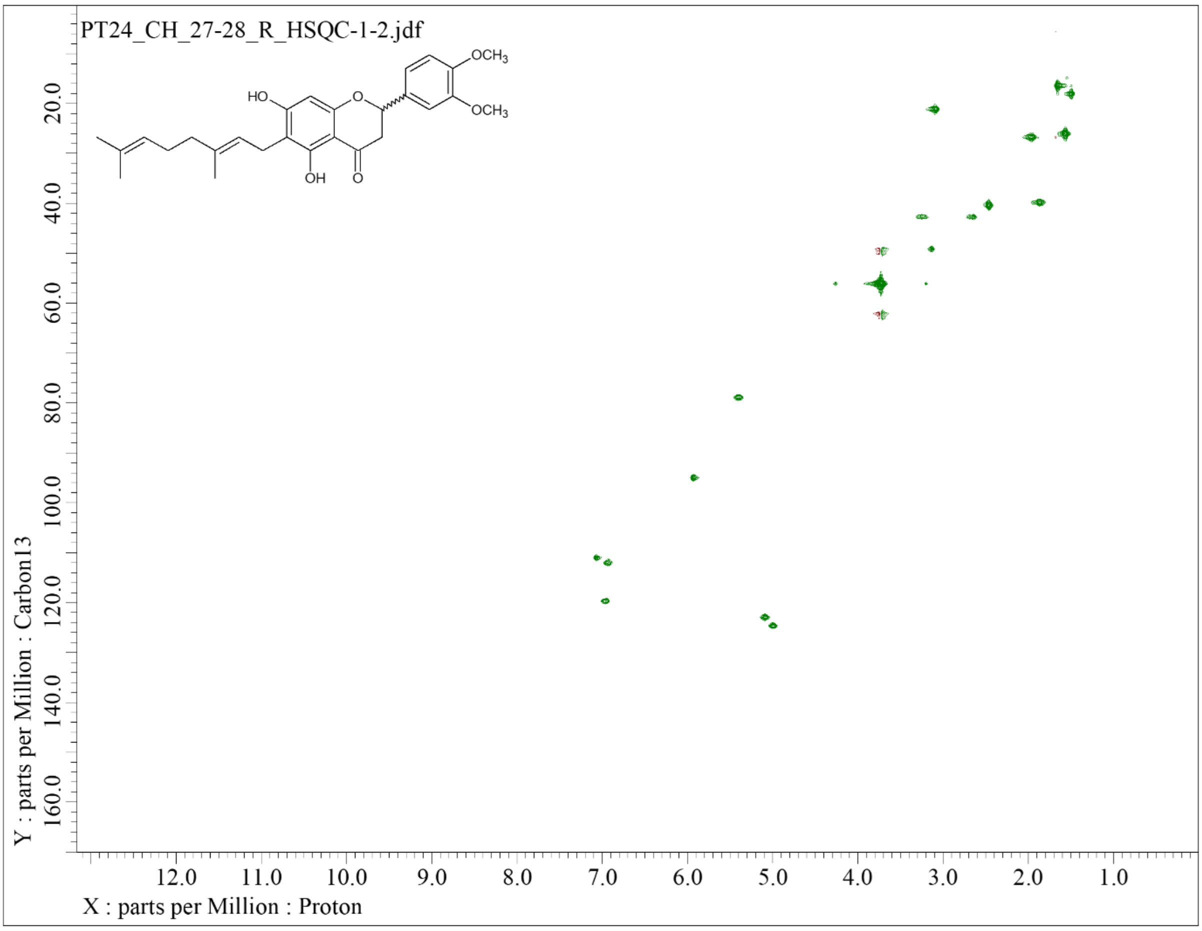

Figure S26. HSQC spectrum (400 MHz, DMSO-d_6) of 3′,4′-O-dimethyldiplacone (**3**)

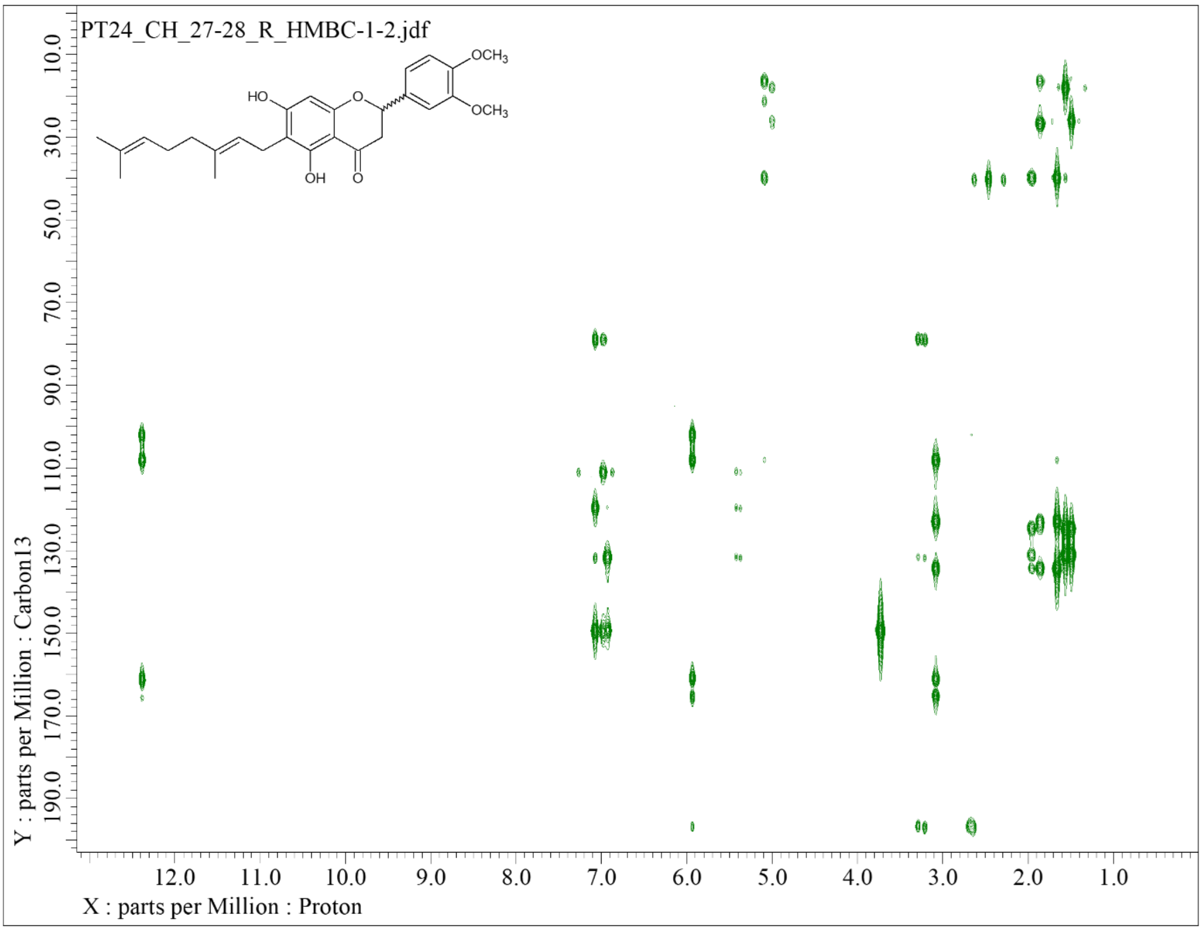

Figure S27. HMBC spectrum (400 MHz, DMSO-d_6) of 3′,4′-O-dimethyldiplacone (**3**)

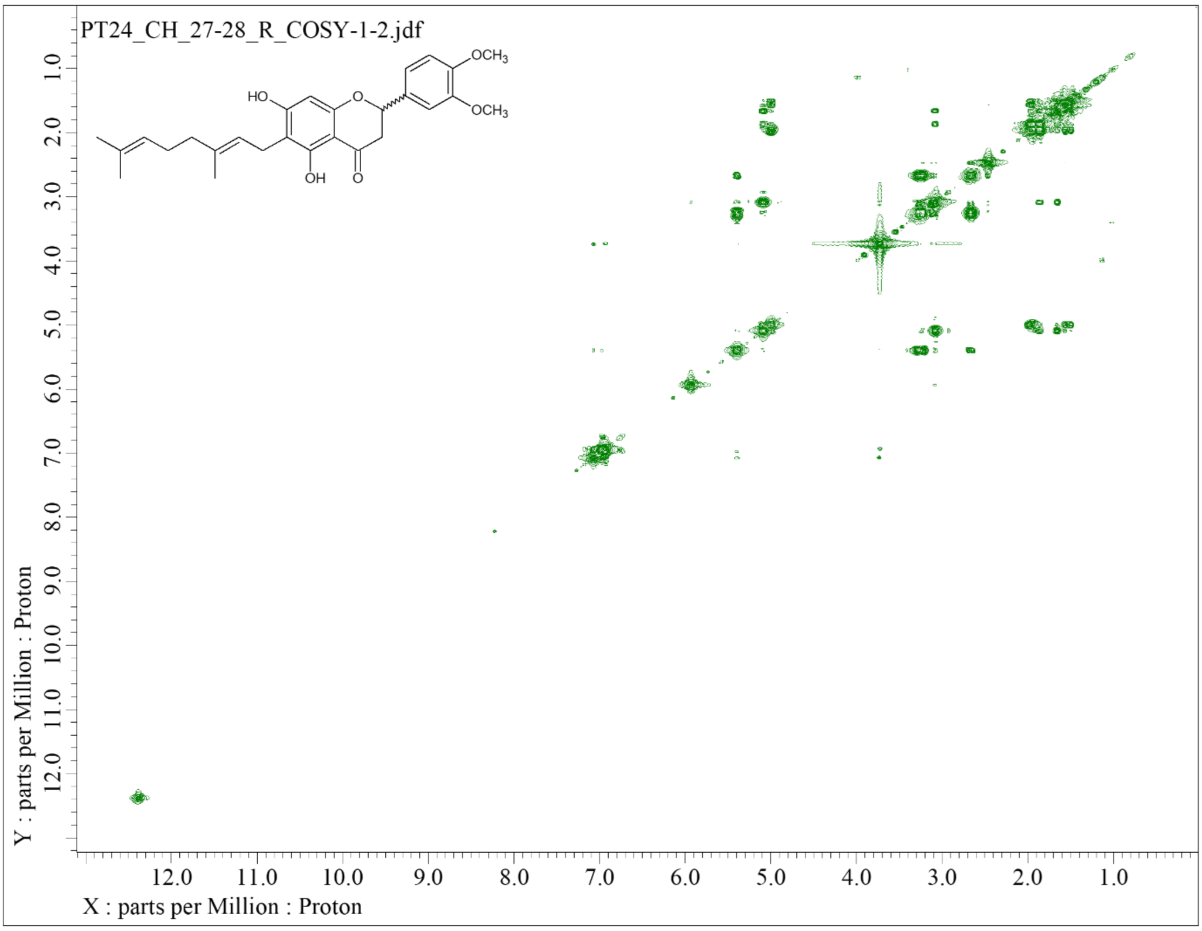

Figure S28. COSY spectrum (400 MHz, DMSO-d_6) of 3′,4′-O-dimethyldiplacone (**3**)

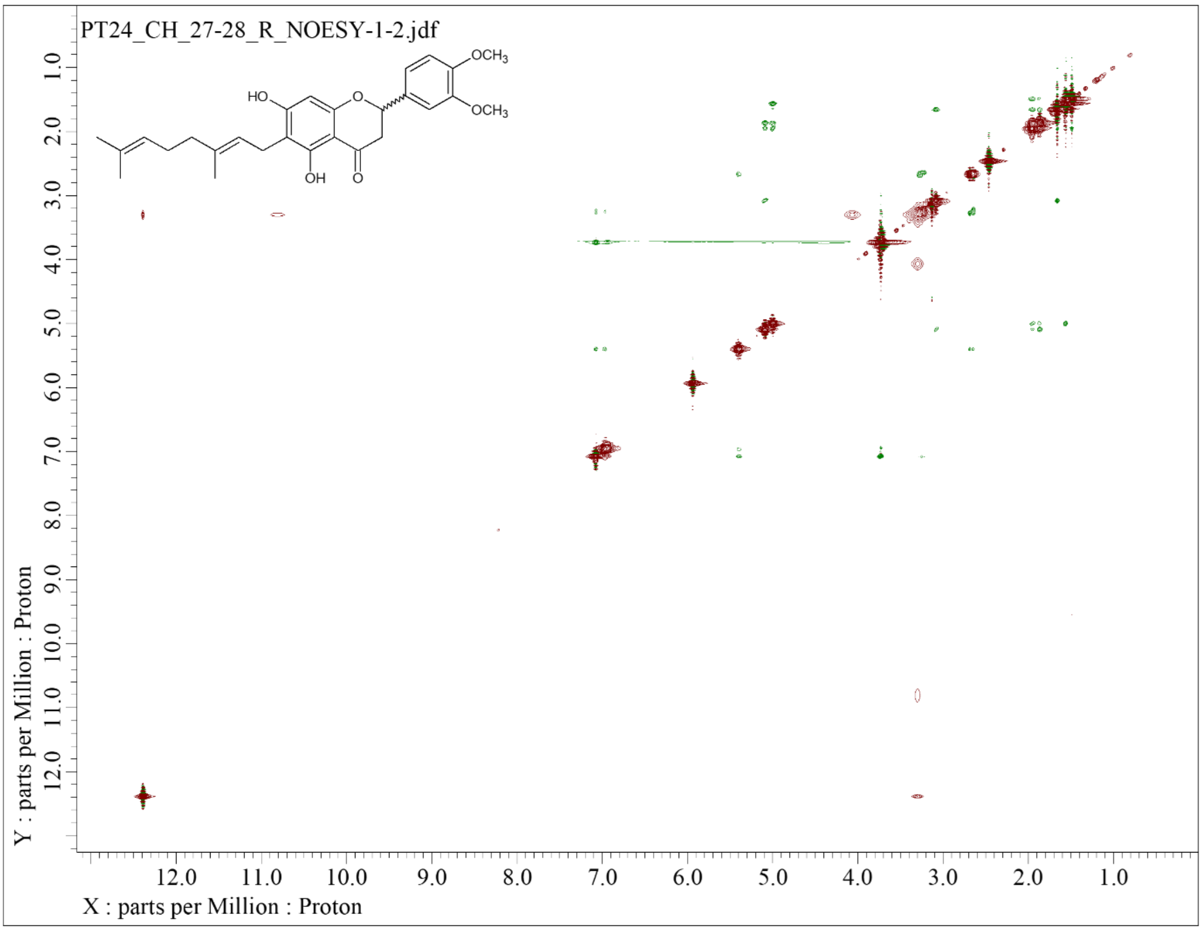

Figure S29. NOESY spectrum (400 MHz, DMSO-d_6) of 3′,4′-O-dimethyldiplacone (**3**)

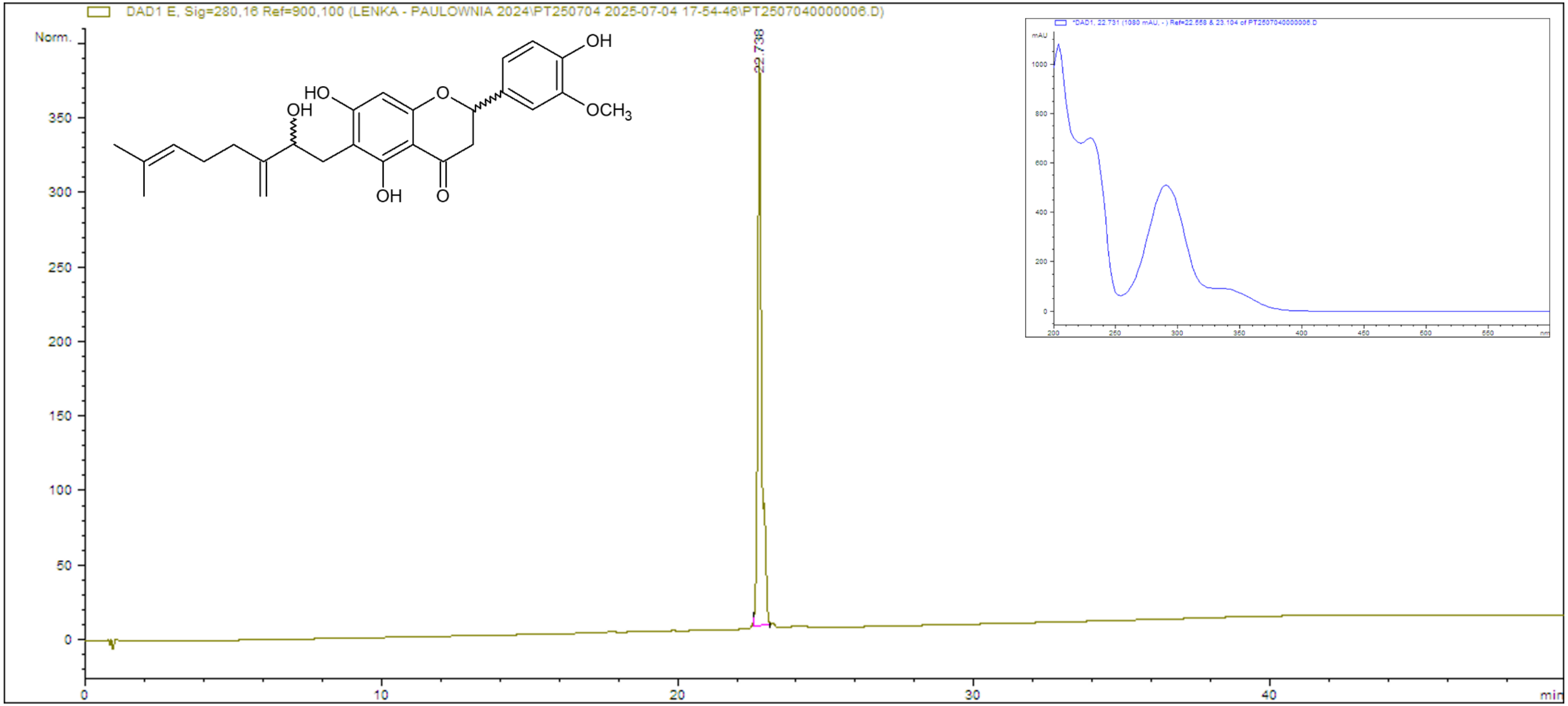

Figure S30. HPLC chromatogram at 280 nm with UV spectrum of tomentodiplacone M (**4**)

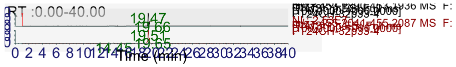

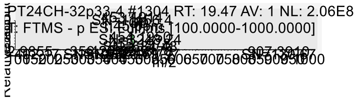

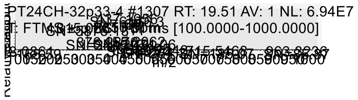

Figure S31. Extracted ion chromatogram (EIC) of the isolated compound **4** from fraction 4 with ID 13504 within 5 ppm *m/z* tolerance and 1% threshold for plot annotations in both negative (upper) and positive polarities (bottom). HRMS spectra at the peak apex in negative (upper) and positive ionization mode (bottom). Identified as tomentodiplacone M (**4**)

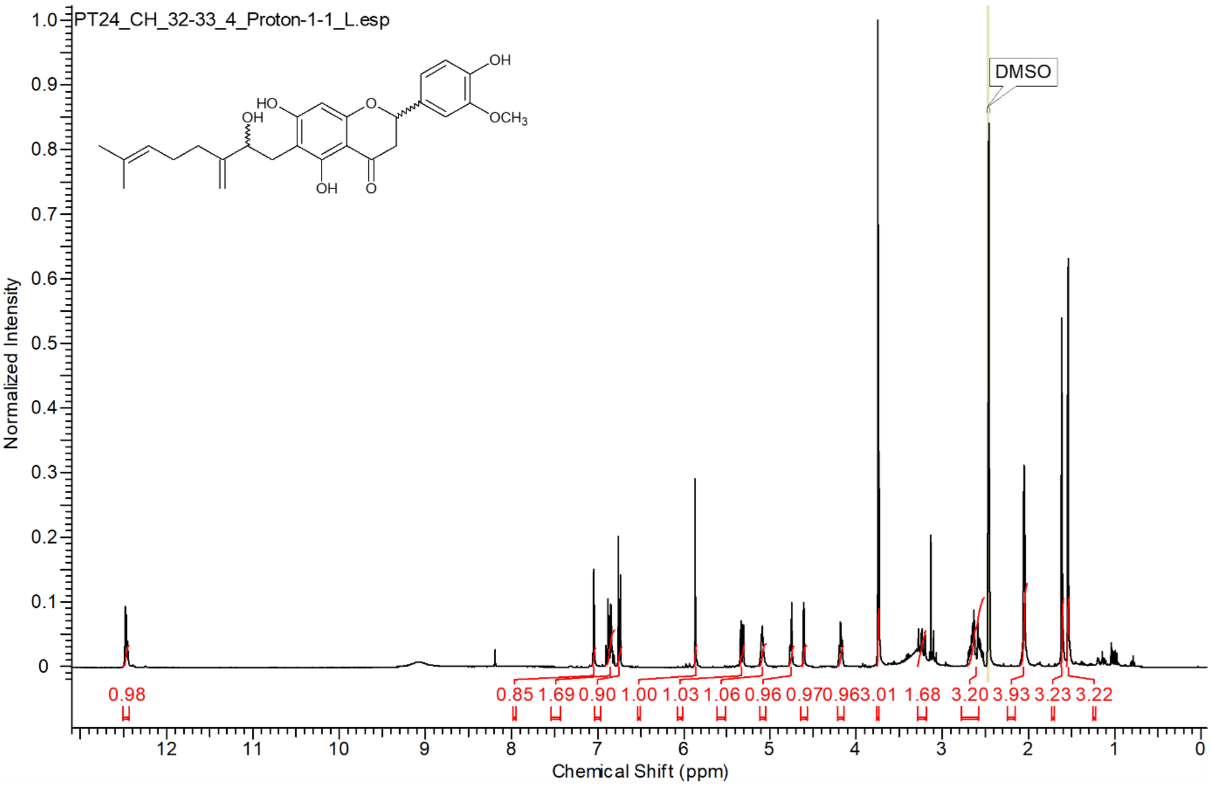

Figure S32. 1H NMR spectrum (400 MHz, DMSO-d_6) of tomentodiplacone M (**4**)

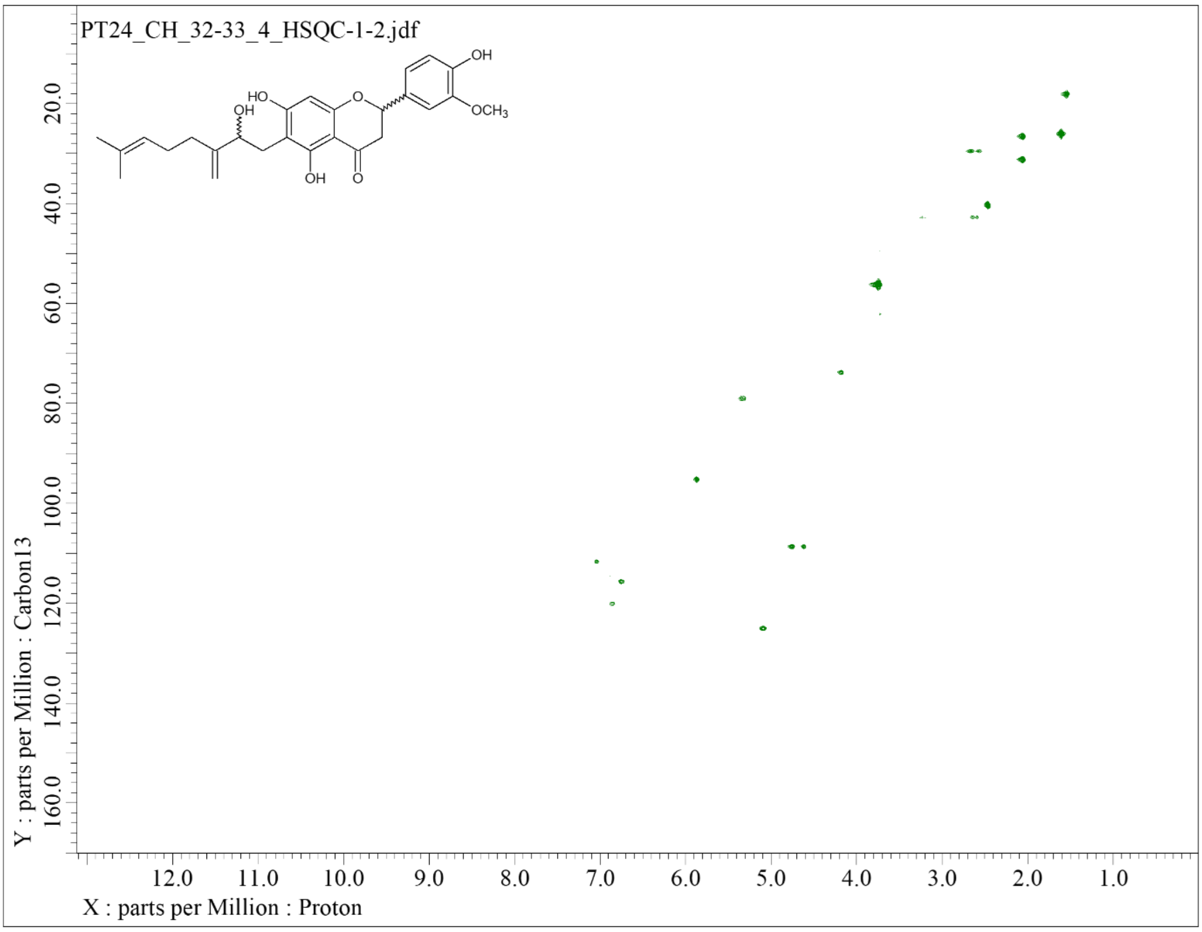

Figure S33. HSQC spectrum (400 MHz, DMSO-d$_6$) of tomentodiplacone M (**4**)

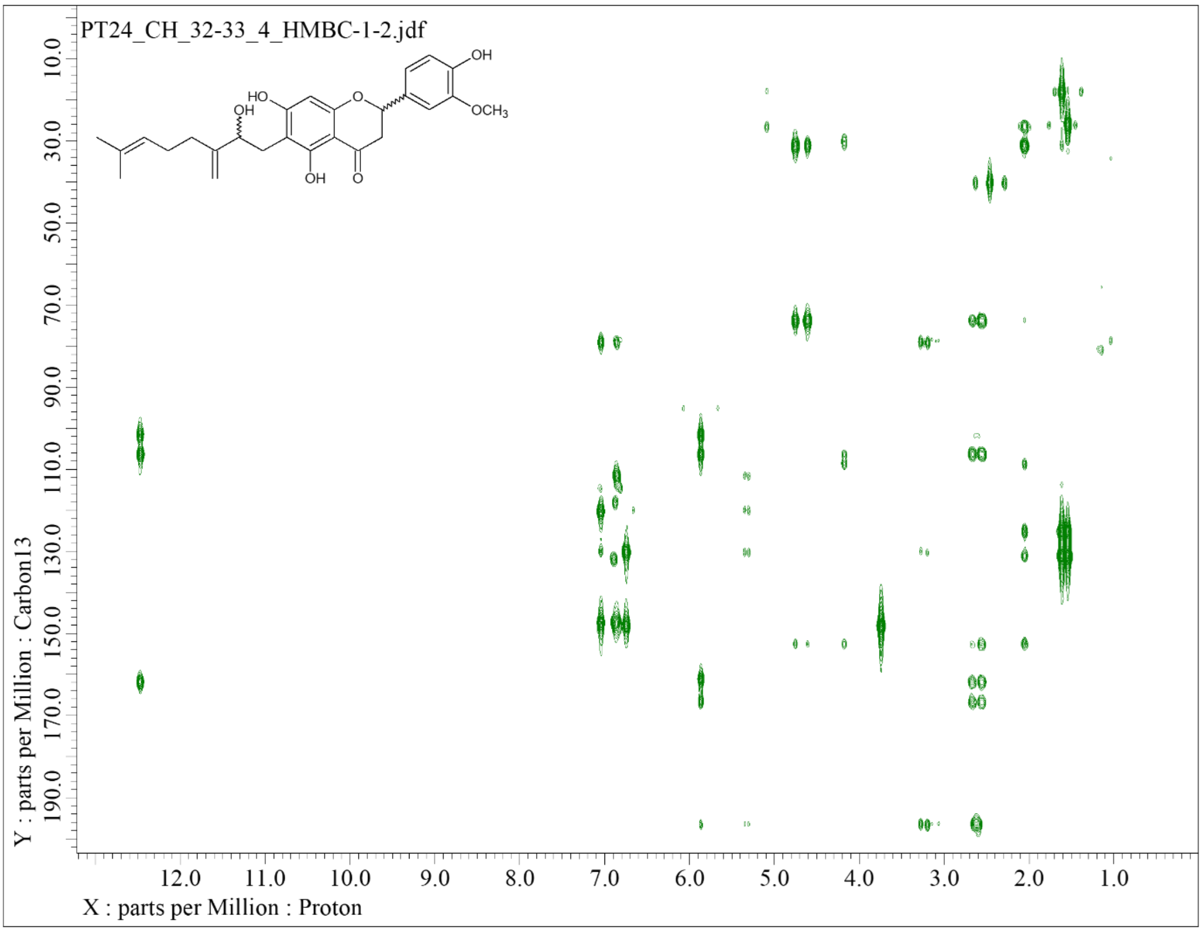

Figure S34. HMBC spectrum (400 MHz, DMSO-d$_6$) of tomentodiplacone M (**4**)

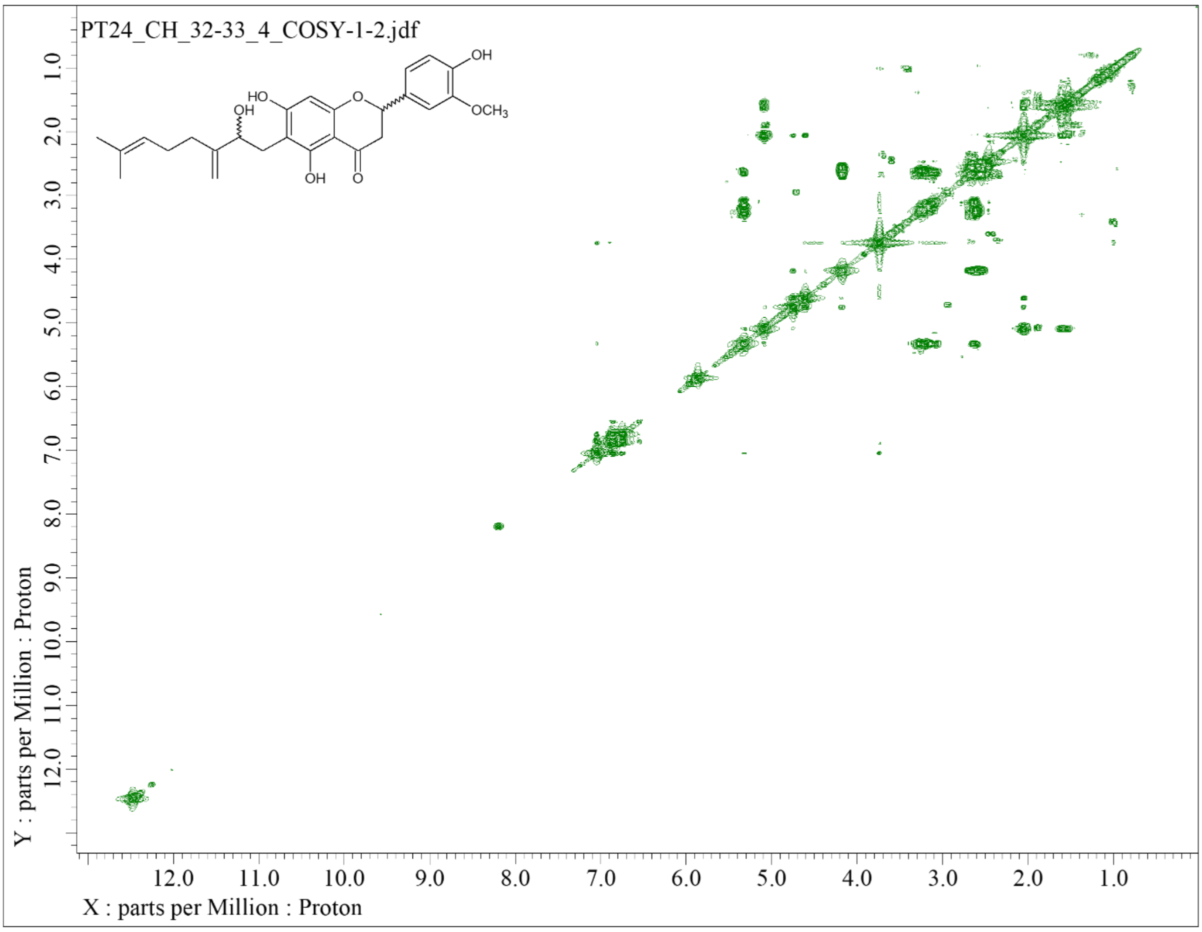

Figure S35. COSY spectrum (400 MHz, DMSO-d$_6$) of tomentodiplacone M (**4**)

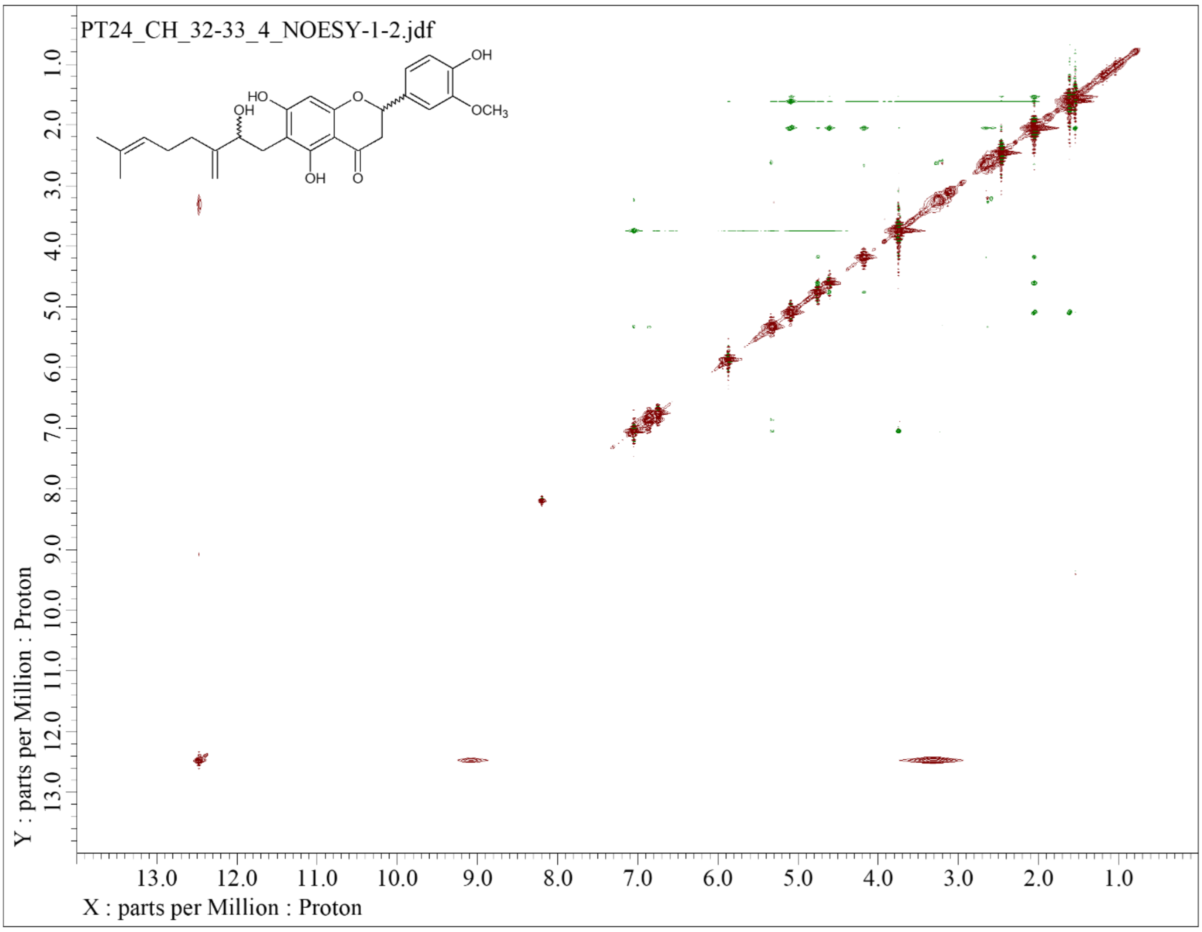

Figure S36. NOESY spectrum (400 MHz, DMSO-d$_6$) of tomentodiplacone M (**4**)

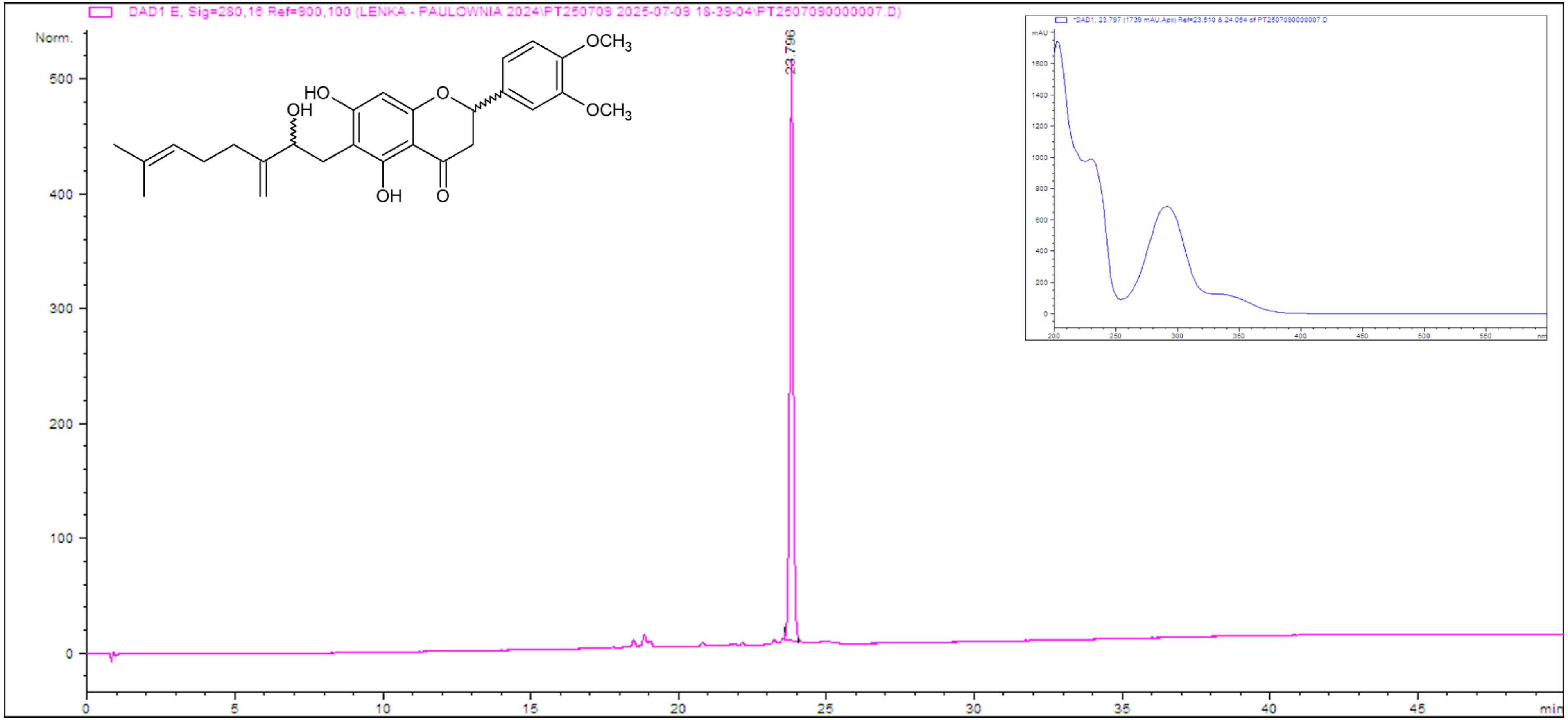

Figure S37. HPLC chromatogram at 280 nm with UV spectrum of 3′,4′-O-dimethylpaulodiplacone A (**5**)

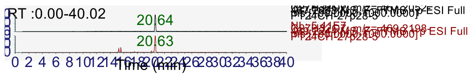

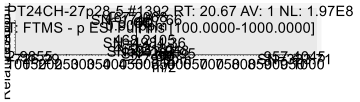

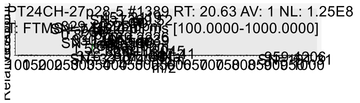

Figure S38. Extracted ion chromatogram (EIC) of the isolated compound **5** from fraction 2 with ID 14313 within 5 ppm *m/z* tolerance and 1% threshold for plot annotations in both negative (upper) and positive polarities (bottom). HRMS spectra at the peak apex in negative (upper) and positive ionization mode (bottom). Identified as 3′,4′-*O*-dimethylpaulodiplacone A (**5**)

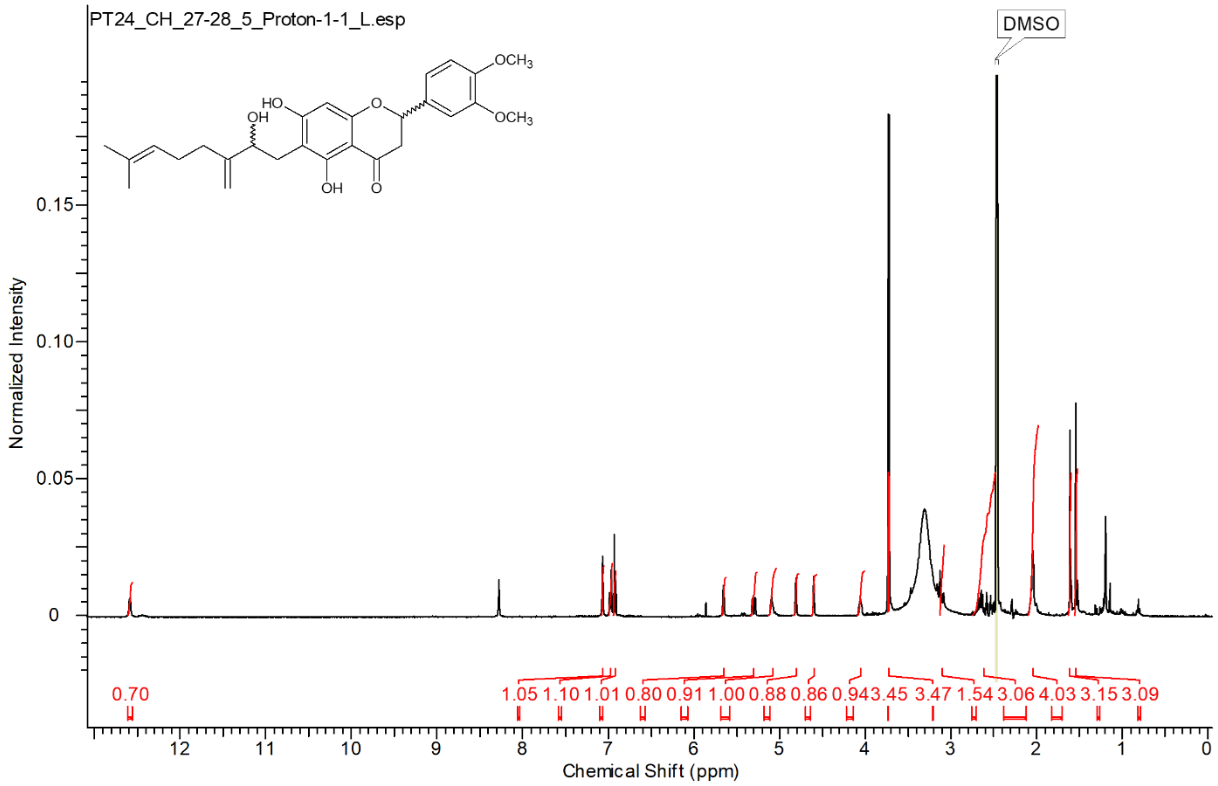

Figure S39. 1H NMR spectrum (400 MHz, DMSO-d_6) of 3′,4′-O-dimethylpaulodiplacone A (**5**)

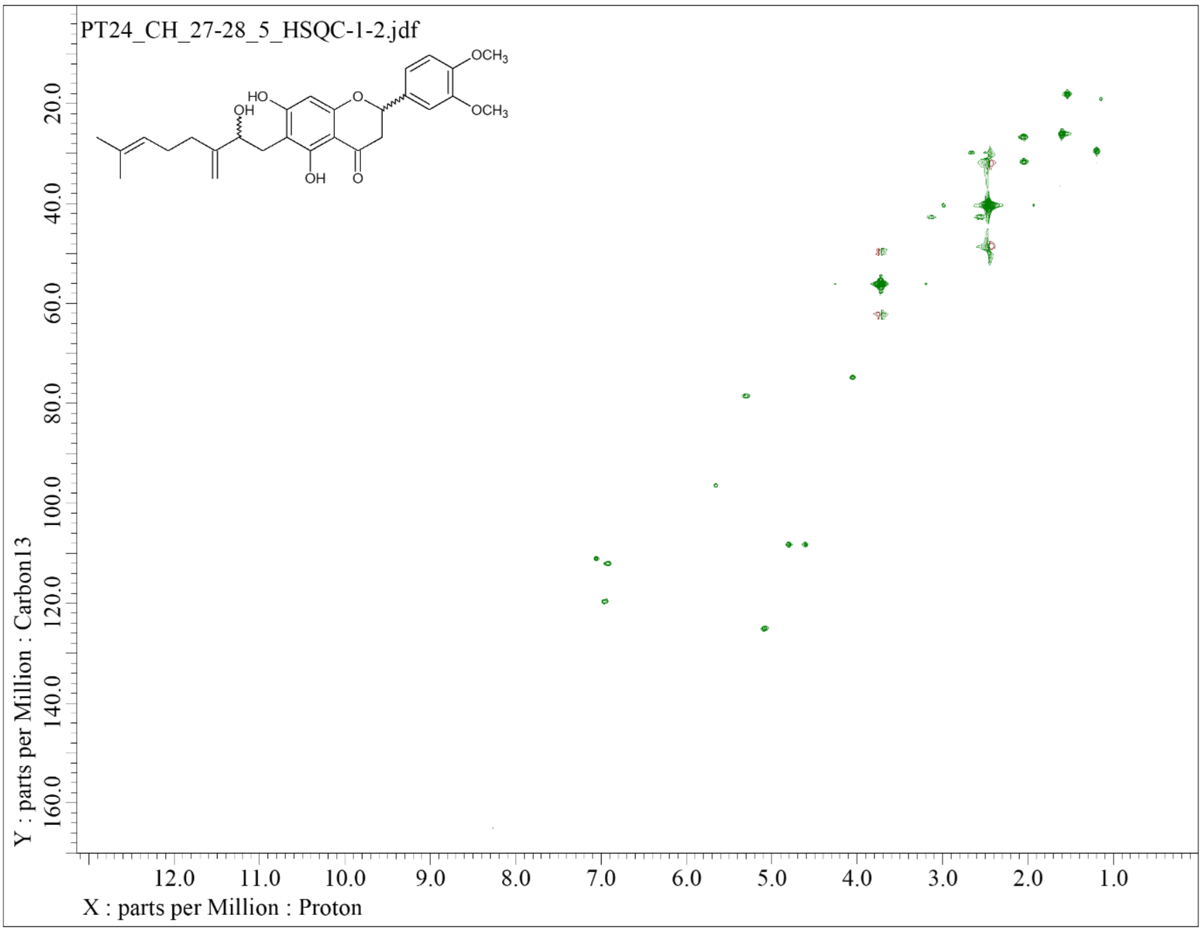

Figure S40. HSQC spectrum (400 MHz, DMSO-d_6) of 3′,4′-O-dimethylpaulodiplacone A (**5**)

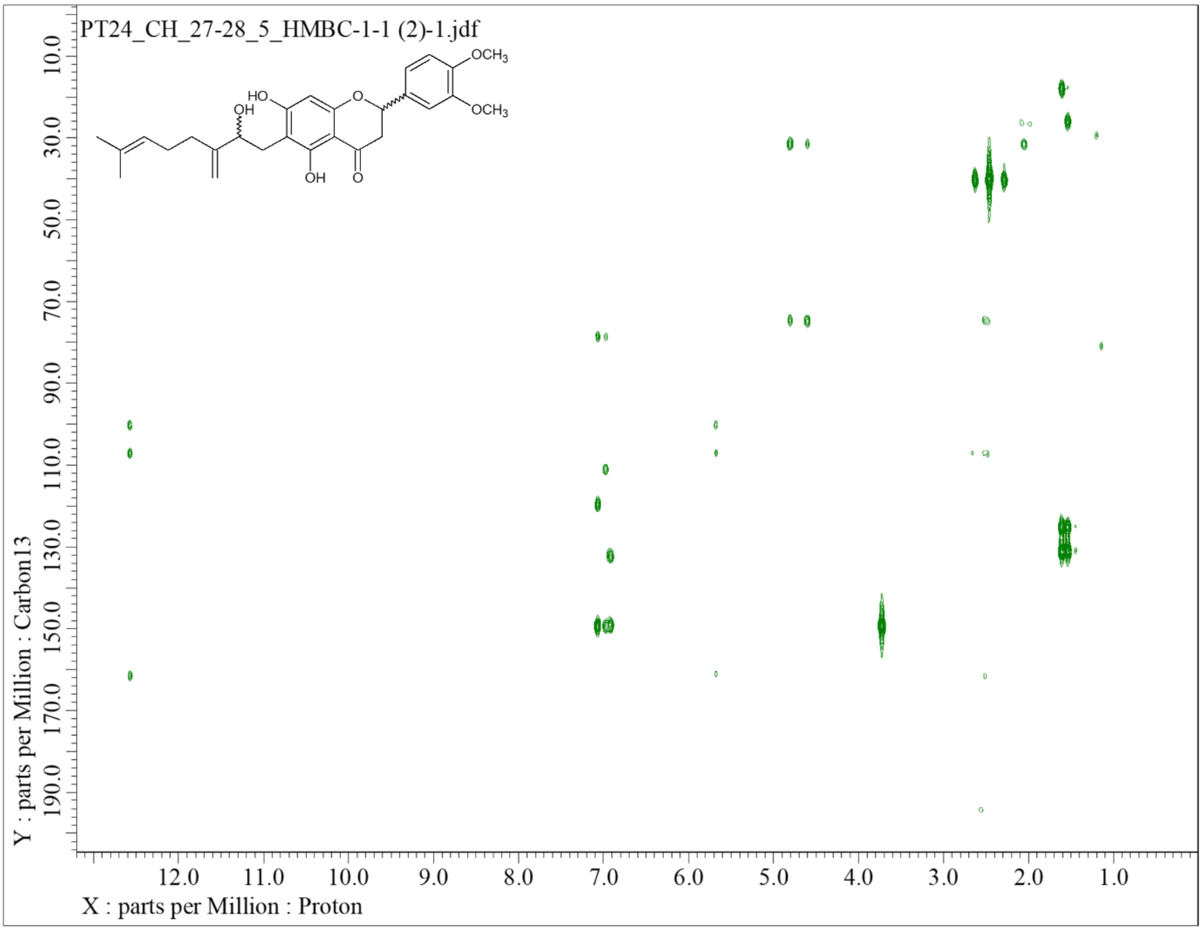

Figure S41. HMBC spectrum (400 MHz, DMSO-d_6) of 3′,4′-O-dimethylpaulodiplacone A (**5**)

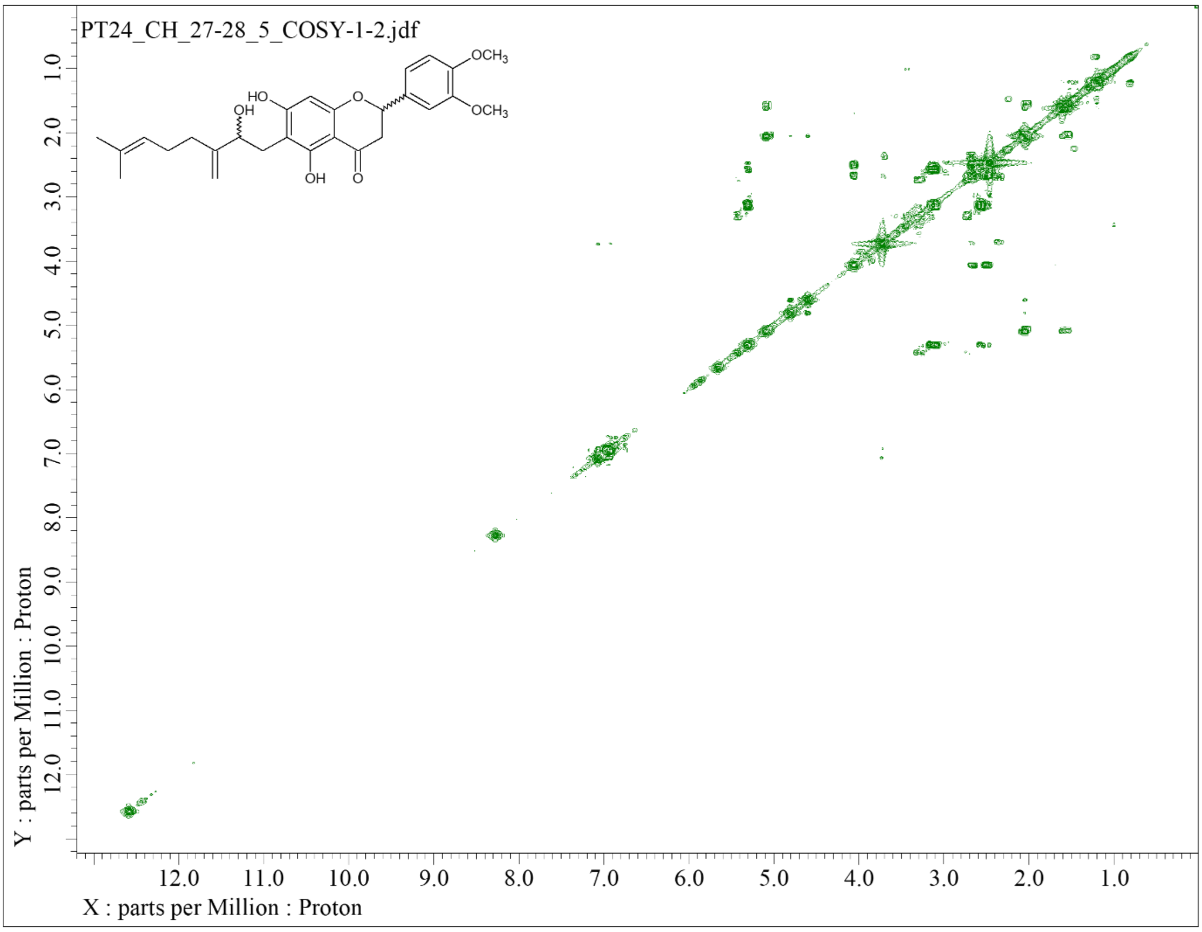

Figure S42. COSY spectrum (400 MHz, DMSO-d_6) of 3′,4′-O-dimethylpaulodiplacone A (**5**)

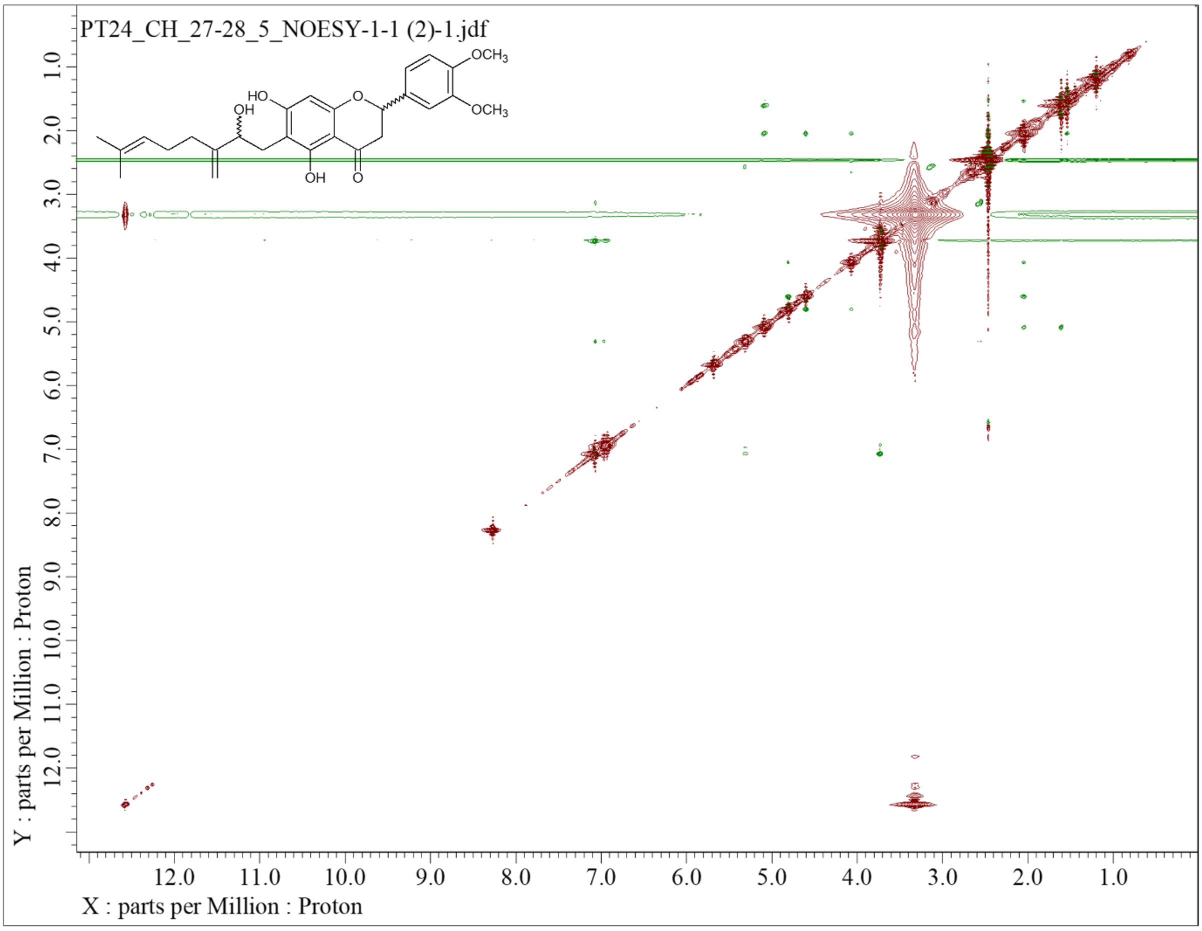

Figure S43. NOESY spectrum (400 MHz, DMSO-d_6) of 3′,4′-O-dimethylpaulodiplacone A (**5**)
